# B3LYP Study on Reduction Mechanisms from O_2_ to H_2_O at the Catalytic Sites of Fully Reduced and Mixed-Valence Bovine Cytochrome *c* Oxidases

**DOI:** 10.1155/2010/182804

**Published:** 2010-04-06

**Authors:** Yasunori Yoshioka, Masaki Mitani

**Affiliations:** Chemistry Department for Materials, Graduate School of Engineering, Mie University, Kurima-machiya 1577, Tsu, Mie 514-8507, Japan

## Abstract

Reduction mechanisms of oxygen molecule to water molecules in the fully reduced (FR) and mixed-valence (MV) bovine cytochrome *c* oxidases (C*c*O) have been systematically examined based on the B3LYP calculations. The catalytic cycle using four electrons and four protons has been also shown consistently. The MV C*c*O catalyses reduction to produce one water molecule, while the FR C*c*O catalyses to produce two water molecules. One water molecule is added into vacant space between His240 and His290 in the catalytic site. This water molecule constructs the network of hydrogen bonds of Tyr244, farnesyl ethyl, and Thr316 that is a terminal residue of the K-pathway. It plays crucial roles for the proton transfer to the dioxygen to produce the water molecules in both MV and FR C*c*Os. Tyr244 functions as a relay of the proton transfer from the K-pathway to the added water molecule, not as donors of a proton and an electron to the dioxygen. The reduction mechanisms of MV and FR C*c*Os are strictly distinguished. In the FR C*c*O, the Cu atom at the Cu_B_ site maintains the reduced state Cu(I) during the process of formation of first water molecule and plays an electron storage. At the final stage of formation of first water molecule, the Cu(I) atom releases an electron to Fe-O. During the process of formation of second water molecule, the Cu atom maintains the oxidized state Cu(II). In contrast with experimental proposals, the K-pathway functions for formation of first water molecule, while the D-pathway functions for second water molecule. The intermediates, P_M_, P_R_, F, and O, obtained in this work are compared with those proposed experimentally.

## 1. Introduction

Cytochrome *c* oxidase (C*c*O) is known to be a terminal oxidase of cellular respiration system and/or electron-transportation system in aerobic organism and to be also a metalloenzyme in inner membrane of mitochondria. It catalyzes the reduction of oxygen molecule to water molecules with the sequential four-electron transfer from cytochrome *c* through heme *a* and it also moves four protons from the matrix side (N-side) of mitochondrial membrane toward the cytosolic side (P-side) (so-called proton pumping) [1–4].

(1) $${\text{O}}_{2}+4{\text{e}}^{-}+8{\text{H}}^{+}\overset{\text{C}c\text{O}}{\to }2{\text{H}}_{2}\text{O}+4{\text{H}}^{+}\;\left(\text{proton}\;\text{pumping}\right).$$

The three-dimensional structures of both fully oxidized (FO) and fully reduced (FR) forms, which are isolated from *Paracoccus* [5, 6] and bovine heart muscle [7–12], have been determined by X-ray crystallographic studies. The catalytic sites of O_2_-reduction of both FO and FR forms are composed of heme *a*_3_ (Fe) and Cu_B_ (Cu) binuclear site. The reduced state of the catalytic site, [Fe(II) Cu_B_(I)], catalyzes the O_2_-reduction, while the oxidized state, [Fe(III) Cu_B_(II)], does not [1–5, 7–13], However, the geometries of the catalytic sites in FO and FR C*c*Os are quite similar. In the bovine heart enzyme, heme *a*_3_ has single ligand of imidazole from histidine residue (His376), and Cu_B_ also has three ligands of imidazoles from histidine residues (His240, His290, and His291) [7–12]. A *ε*-nitrogen of His240 is uniquely cross-linked to C6 of phenol of tyrosine (Tyr244) with single covalent bond. The heme-copper oxidases which have been determined by the X-ray crystallographic analyses [7, 8, 14–16] have a common structure to the bovine C*c*O. This superfamily has been classified into A1, A2, B, and C families by amino acid sequence analyses [17, 18]. The bovine heart C*c*O, which belongs to the A1 family, has two distinct proton pathways, K-pathway and D-pathway [5, 8, 17–23]. The K-pathway begins from Lys319 and ends at Thr316, while the D-pathway begins from Asp91 and ends at Glu242. The K-pathway is used to transfer two protons toward the catalytic site, while the D-pathway is used to transfer the remaining six protons. Thus, the D-pathway transfers both two substrate protons to reduce the O_2_ molecule and four protons that are pumped across the membrane [24, 25]. It is presumed that the branching point is located at Glu242.

As can be seen in Table 1, the mixed-valence (MV) C*c*O and FR C*c*O should be strictly distinguished. The FR state has four electrons to produce two H_2_O molecules due to the reduced valence state of Cu_A_, heme *a*, heme *a*_3_, and Cu_B_, while the MV state has only two electrons in the catalytic site of heme *a*_3_ and Cu_B_. Thus, the MV state has possibility to produce only one H_2_O molecule. Although both MV and FR states have two electrons in the catalytic site of heme *a*_3_ and Cu_B_, it is expected that the reduction mechanisms of the O_2_ molecule for MV and FR C*c*Os should be different after the [Fe(III)-O_2_^−^  Cu(I)] intermediate was formed. The [Fe(III)-O_2_^−^ Cu(I)] intermediate of FR C*c*O have possibility to receive an electron from heme *a*, while an electron in Cu(I) should be used to reduce the O_2_ molecule in MV C*c*O.

**Scheme 1:**
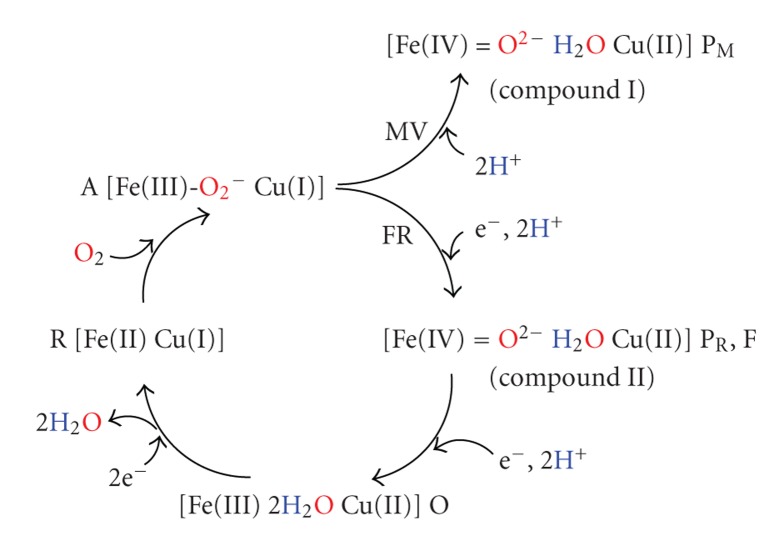
Schematic representation of pathways of O_2_ reduction to produce H_2_O at the catalytic sites of MV and FR C*c*Os.

The numerous mechanisms of the reduction of O_2_ molecule to H_2_O molecule have been proposed experimentally [2–4, 13, 17, 19–22, 26–55]. There is now consensus that O_2_ molecule in the triplet state is initially bound to Fe atom of heme *a*_3_ in the reduced state [Fe(II) Cu(I)] (R) to yield the ferric peroxide intermediate [Fe(III)-O_2_^−^ Cu(I)] (A), as shown in Scheme 1. The intermediate A has the optical absorbance at 595 nm and a mode of 568 cm^−1^ due to the Fe-O_2_ vibration was detected by resonance Raman studies [2, 3]. The subsequent intermediate [Fe(IV)=O^2−^ H_2_O Cu(II)], which includes Fe(IV)=O^2−^ in heme *a*_3_ as shown in Scheme 1, has also been experimentally observed [3, 19, 20, 26–28, 31–40]. [Fe(IV)=O^2−^ H_2_O Cu(II)] is usually designated by the symbols, P_M_, P_R_, and F [2, 17, 19, 33–36]. It is considered that the differences of these intermediates are due to the protonation state of a nearby protonable center or number of electrons in the catalytic site. For MV C*c*O, a mode of 804 cm^−1^ due to Fe=O vibration has been observed by resonance Raman spectroscopy [31], while for FR C*c*O a mode of 786 cm^−1^ has been observed [41]. Before formation of the intermediate F, it was shown from both optical and EPR spectroscopy that the P_R_ intermediate exists and exhibits spectroscopic properties quite distinct from F [30, 32, 42].

However, the reaction mechanism from the [Fe(III)-O_2_^−^ Cu(I)] (A) to [Fe(IV)=O^2−^ H_2_O Cu(II)] (P or F) is not conclusive yet. Although the hydrogenated/protonated Fe-OOH, which will be considered as the intermediacy from A to P or F, has been discussed in numerous proposals based on the experimental results, it is not beyond the region of speculation. In addition, the geometrical and electronic structures of [Fe(IV)=O^2−^ H_2_O Cu(II)] and Fe-OOH have not been entirely elucidated yet. Yoshikawa and coworkers have proposed the mechanism that the proton transfer is induced from Tyr244 to FeOO to yield hydroperoxide and subsequently one electron transfer from Cu_B_ is induced to cleave O–O bond [9, 55]. [Fe(IV)=O^2−^ OH^−^-Cu(II)] is produced with tyrosyl radical and anion for MV and FR C*c*O, respectively. [Fe(IV)=O^2−^ OH^−^-Cu(II)] is supported as an intermediate at the next stage from the intermediate A in lots of experimental examination [21, 22, 32, 43, 44]. The Cu atom plays a role for electron storage and changes in its oxidation state [36]. It has been proposed for FR C*c*O that the oxidation states of Cu and Fe atoms in heme *a* and heme *a*_3_ change through the reaction without generation of oxoferryl-tyrosine radical intermediate which was formed in the MV C*c*O [21, 22]. It has been also proposed by Wikström that phenol of tyrosine dose not affect the reaction [32]. In FR C*c*O, Fe of heme *a* (Fe*_a_*) is initially in the ferrous state. From optical experiments, Fe*_a_* is oxidized at the same time that the [Fe(III)-  O_2_^−^ Cu(I)] intermediate disappeared [45, 46]. This observation is also supported from resonance Raman experiments and it was concluded that the electron transfers from Fe*_a_* to binuclear center [47]. Several groups have speculated that the crosslinked tyrosine plays roles for a hydrogen atom donor [2, 21, 29, 31, 48] to molecular oxygen bound to heme *a*_3_ in order to activate O–O bond. It has been proposed from recent experimental studies that a tyrosyl radical is formed in the [Fe(IV)=O^2−^ H_2_O Cu(II)] intermediate [1–4, 31, 49, 50]. Direct evidences are not, however, observed. The mutation of histidine coordinated to Cu induced the damage of catalytic effects with retaining the electron transfer between heme *a*_3_ and heme *a* [51–54]. The reaction mechanisms proposed by several groups are still controversial and the structures of the [Fe(IV)=O^2−^ H_2_O Cu(II)] intermediates, P_M_, P_R_, and F and the intermediacy Fe-OOH are still unknown and their changes through O_2_-reduction are also unknown.

The reaction proceeds in a stepwise manner by the transfer of four electrons and four protons. There are K- and D-pathways for the proton transfer [44, 56–63]. The D-pathway that ends at Glu242 near to the catalytic site has been experimentally and theoretically studied. The molecular dynamic simulations have shown that the conformational switch of Glu242 functions the proton pumping through H_2_O network connecting to the D-propionate group of heme *a*_3_ and transfer of two protons through H_2_O network connecting to the catalytic site [57, 58]. The FTIR measurement has shown that the reduction of O_2_ molecule stops at the P_R_ intermediate in the Glu278Gln mutant enzyme from *paracoccus denitrificant* [59]. It was also proposed that the K-pathway is catalytic only in the last steps of the catalytic cycle [44, 60, 61]. It was, however, proposed that the K-pathway is used for the uptake of two substrate protons upon reduction of catalytic site [23, 24, 60]. Recently, Lepp et al. showed that mutations in the K-pathway of proton transfer slowed down formation of the P_R_ intermediate [64]. Thus, the sequential uptakes of four substrate protons from the K- and D-pathways are still unclear.

On the other hand, theoretical studies on the reduction mechanism and the proton pathways in C*c*O have been extensively performed [65–92]. “Splitting the Water Molecule” mechanism has been proposed based on the hybrid density functional calculations [65–68]. The water molecule is initially located in the vicinity of the Cu_B_ center in their mechanism. This water molecule provides a proton to oxy intermediate [Fe(III)-  O_2_^−^ Cu(I)] and at the same time copper atom provides an electron. The products of this mechanism would be [Fe(III)-OOH HO^−^-Cu(II)]. The reaction systems were calculated on the potential energy surface of the triplet state. For MV C*c*O in which an electron cannot transfer from heme *a* to heme *a*_3_, the density functional theory (B3LYP) has been applied to examine O–O bond cleavage using a large model of Fe(II)-Cu(I) binuclear site [69–71]. They have proposed that it is necessary to add two water molecules in the catalytic site in order to form hydrogen bonds connecting between Fe-OO and Tyr244. It was also proposed that the proton transfer from the K-pathway to the catalytic site enhances the proton transfer from Tyr244 to FeOO. In recent works [71, 72], they employed the bigger model that contains the Cu_B_ moiety, Tyr244 and protonated lysine. It was proposed that the protonation of the FeOO proceeds the OO bond cleavage with higher activation energy of 18.6 kcal/mol than the experimental value and yields the [Fe=O, HO-Cu] intermediate where the tyrosine is a neutral radical. Namely they showed that the additions of two electrons and single proton to the OO bond induce the OO bond cleavage to yield the P_M_ intermediate.

In our previous works [76, 77], we have pointed out the possibility of existence of single water molecule between His290 and Tyr244 with the hydrogen bonds. For the heme-dioxygen complex [78, 80], we have shown that the OO bond cleavage occurs when the OO bond receives two electrons and two protons. For the H_2_O formation in FR C*c*O [79], we showed that the H_2_O molecule on the Cu atom plays crucial roles. This H_2_O molecule provides a proton to Fe(IV)=O^2−^ to yield [Fe(III)OH HOCu(II)] with the electron transfer from Cu_B_ to heme *a*_3_, so called the proton-coupled electron transfer. From these results, we have also speculated the mechanism of the H_2_O formation for FR and MV C*c*O. However, the reduction mechanism and catalytic cycle were not studied systematically and concretely.

In this article, we propose new reduction mechanisms from O_2_ molecule to H_2_O molecules by MV and FR C*c*Os from theoretical viewpoints. The intermediates, and their electronic structures obtained by the sequential additions of electrons and protons are thoroughly examined. The functions of Tyr244 in our mechanism are distinguishably different from those proposed from experiments as a proton and an electron donor. This paper is composed of as follows: (1) possibility of H_2_O coordination to Cu of the Cu_B_ site is examined, (2) formation mechanism of single H_2_O molecule from [Fe(III)-  O_2_^−^ Cu(I)] (A) in MV C*c*O is examined, (3) formation mechanism of two H_2_O molecules from [Fe(III)-  O_2_^−^ Cu(I)] (A) in FR C*c*O is examined, (4) the reduction mechanisms for MV and FR C*c*O are summarized. Our reaction scheme is compared with other mechanisms proposed previously from experimental and theoretical viewpoints.

## 2. Computational Details

### 2.1. Model of a Catalytic Site for Calculations

**Figure 1:**
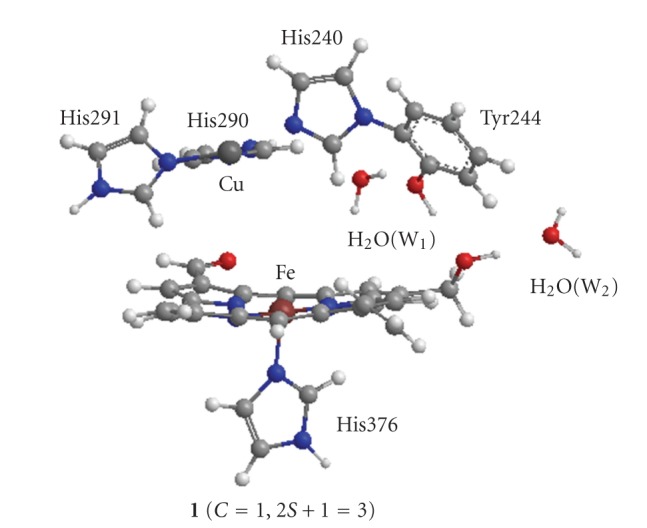
Model of catalytic site in fully reduced form of bovine heart cytochrome *c* oxidase (1OCR in PDB). The added two water molecules W_1_ and W_2_ are detailed in text.

The model of the catalytic site of C*c*O to examine the O_2_ reduction mechanism was constructed from geometry based on the X-ray crystallographic study for FR C*c*O of bovine heart muscle (1OCR in PDB) [9]. As shown in Figure 1, all histidine residues, His240, His290, His291, and His376, were replaced by imidazoles. Tyr244, which is covalently bonded to His240, was replaced by phenol. The formyl and vinyl groups in heme *a*_3_ were left on the porphyrin ring due to the possibility of the *π*-resonance. The farnesylethyl group was replaced by –CH_2_OH due to the possibility to make hydrogen bond to phenol (Tyr244).

We added two water molecules, W_1_ and W_2_, in this model. In the X-ray crystallographic studies [10–12], single H_2_O molecule is found between –CH_2_OH of farnesylethyl group and Thr316 that is a terminal residue of the K-pathway starting from Lys319. The added W_2_ corresponds to this H_2_O molecule. We do not examine the proton transfer from Thr316 to –CH_2_OH through W_2_ in this study. However, we examine explicitly the intermediates where a proton attaches on –CH_2_OH. Thus W_2_ is added into the model.

Although W_1_ is not shown in the X-ray structures, there is a possible space that a H_2_O molecule fit into between Tyr244 and His290. Particularly, it can be seen in Figure 1 that W_1_ is hydrogen-bonded to both Tyr244 and His290. By adding this W_1_, the network of the hydrogen bonds of the K-pathway is expanded from Thr316 to W_1_ through W_2_, farnesylethyl, and Tyr244.

### 2.2. Theoretical Examination

The Fe and Cu atoms have possibilities of unpaired electron spins for the oxidation states of Fe(III) and Cu(II) in the course of reduction of the O_2_ molecule, respectively. Their unpaired spins will be ferromagnetically coupled in the high-spin (HS) state, while they are antiferromagnetically coupled in the low-spin (LS) state. The electronic structure of the HS state can be well presented by a usual unrestricted molecular orbital method. The LS states can be presented by the unrestricted SCF solutions with the broken symmetry (BS) procedure. The all-electron DZ basis set was employed for Fe and Cu atoms [93]. The 6-31G* basis set was used for key O atoms of reacting O_2_ molecule, phenol and H_2_O molecules and the 3-21G basis set for C, H, N, and other O atoms.Although the 3-21G basis set is tight for the transition-metal complexes in some cases, these combined basis sets used here reproduced reasonably the electronic structures of heme-oxygen complexes estimated by using more flexible basis sets [80].

Since correlation effects are important to elucidate the transition-metal systems, the usual Hartree-Fock methods lead to poor estimations for the binuclear systems. The hybrid exchange-correlation functional B3LYP method [94–97] was most widely used for the transition-metal system. Since the B3LYP method contains the moderate static correlation effects, it provided the suitable results for the desired d-electron configurations in good agreement with experiments [80, 98, 99]. Thus, we employed the B3LYP method to estimate the electronic structures of the reaction systems. 

The dioxygen binds to Fe in heme *a*_3_ at an initial stage of the reaction. The bound dioxygen is reduced by sequential additions of four protons and four electrons on heme *a*_3_. Four electrons transfer from Cu_A_ to the active site through heme *a*. Thus the examination of the reaction mechanism is equivalent to determining the pathway to provide the protons to the dioxygen on heme *a*_3_. The conformation of the catalytic site in the FR C*c*O is similar to that in the FO state [7–12]. Heme *a*_3_, Cu, His240, His290, His291, and Tyr244 have same geometrical configurations for both FR and FO C*c*O. The Cu atom is fixed by coordination of three histidine residues, His240, His290 and His291. Tyr244 makes the hydrogen bond to the farnesylethyl, group in heme *a*_3_. Tyr244 is fixed by its hydrogen bond and the cross-linked single covalent bond with His240 that coordinates to the Cu atom. Heme *a*_3_ is also fixed by the axial coordination of His376 and the hydrogen bond with Tyr244. Accordingly the essential change for the structure of the active site is not expected in the reduction of the dioxygen on heme *a*_3_. However, the pathway of the proton transfer plays crucial roles. The proton pathway must approach to the dioxygen bound to heme *a*_3_ in order to provide protons. The water molecule W_1_ connects with the K-pathway through the hydrogen bonds of W_1_, Tyr244, farnesylethyl and W_2_. In fact, in our previous work [76], the hydronium ion W_1_H^+^, where a proton is added to W_1_, approaches to the dioxygen to give the proton, yielding the bond FeOOH on the heme *a*_3_. Thus it is expected that a remarkable change is found in the proton pathway of the hydrogen bond network. The fragments of reacting O_2_, H_2_O molecules, and H atoms of –CH_2_OH and OH in phenol, which are directly connected with the hydrogen bond network, were optimized. Since our optimizations were performed for limited parameters, our discussion will be qualitative not quantitative. We draw the potential energy surfaces along the path of the proton transfer in a stepwise manner. It could be confirmed form the potential energy surfaces that the optimized intermediates are local minimums. The point with maximum energy is assigned to the transition state because of limited optimization. However, we believe that our transition states are close to the fully optimized one and the relative stabilities among the intermediates and transition states are qualitatively reliable. All calculations were carried out using the program package Gaussian 98 [100].

### 2.3. Analyses for BS Solutions

The system examined here is composed of the open-shell chemical species, since the two transition metals Fe and Cu have the unpaired electron spins. The spin-unrestricted calculations are employed to describe the electronic structures. Particularly, the broken symmetry (BS) method is used for the LS states. It is well known that the BS solutions are suffered by the spin contamination ${\langle {\hat{S}}^{2}\rangle }_{\text{SC}}$. However, ${\langle {\hat{S}}^{2}\rangle }_{\text{SC}}$ is related with the occupation numbers of electron in the natural orbitals that are obtained by the diagonalization of the first-order density matrix of the BS solution [80, 99, 101]:

(2) $$\begin{array}{c} {\langle {\hat{S}}^{2}\rangle }_{\text{SC}}={\langle {\hat{S}}^{2}\rangle }_{\text{BS}}-{\langle {\hat{S}}^{2}\rangle }_{\text{Pure}}=\overset{N_{\beta }}{\underset{i=1}{\sum }}n_{-i}n_{+i}≅\overset{N_{p}}{\underset{i=1}{\sum }}n_{-i}n_{+i} \end{array},$$

(3) $$n_{-i}+n_{+i}=2,\;1\leq n_{-i}\leq 2.$$

Here, *n*_−*i*_ and *n*_+*i*_ are the occupation numbers of the bonding natural orbitals *ϕ*_−*i*_ and antibonding *ϕ*_+*i*_, respectively. *N*_*β*_ is the number of *β*-electron. ${\langle {\hat{S}}^{2}\rangle }_{\text{BS}}$ is the expectation value of square of the spin angular momentum for the BS solution, while ${\langle {\hat{S}}^{2}\rangle }_{\text{Pure}}$ is one of the corresponding pure spin state. When *ϕ*_−*i*_ is an doubly occupied orbital, it does not contribute to the spin contamination because of *n*_−*i*_ = 2 and *n*_+*i*_ = 0. For the BS solution where *ϕ*_−*i*_ and *ϕ*_+*i*_ are coupled antiferromagnetically, the spin contamination ${\langle {\hat{S}}^{2}\rangle }_{\text{SC}}$ is increased by unity because of *n*_−*i*_ ≃ *n*_+*i*_ ≃ 1 and *n*_−*i*_ *n*_+*i*_ ≃ 1. In the BS calculation of the singlet state, if the single pair of the antiferromagnetic spin coupling exists in the system, the ${\langle {\hat{S}}^{2}\rangle }_{\text{BS}}$-value will be nearly equal to unity with ${\langle {\hat{S}}^{2}\rangle }_{\text{Pure}}=0$. For the BS calculation of the doublet state, ${\langle {\hat{S}}^{2}\rangle }_{\text{BS}}≃1.75$ with ${\langle {\hat{S}}^{2}\rangle }_{\text{Pure}}=0.75$. In other word, the spin contamination ${\langle {\hat{S}}^{2}\rangle }_{\text{SC}}$, the deviation of ${\langle {\hat{S}}^{2}\rangle }_{\text{BS}}$ from ${\langle {\hat{S}}^{2}\rangle }_{\text{Pure}}$ represents the numbers of pairs of the antiferromagnetically coupled spins (*N_p_*) in the system under examination. Although the spin contamination ${\langle {\hat{S}}^{2}\rangle }_{\text{SC}}$ gives valuable information, the coupled spin-site in the system must be identified by the spin population.

The spin contamination is a serious problem in the BS solution. No exact procedure to remove the spin contamination is proposed at the B3LYP level. All procedures proposed currently are approximate. In this paper, we employed the energies for discussion without projection to the pure spin state.

## 3. Results and Discussion

**Figure 2:**
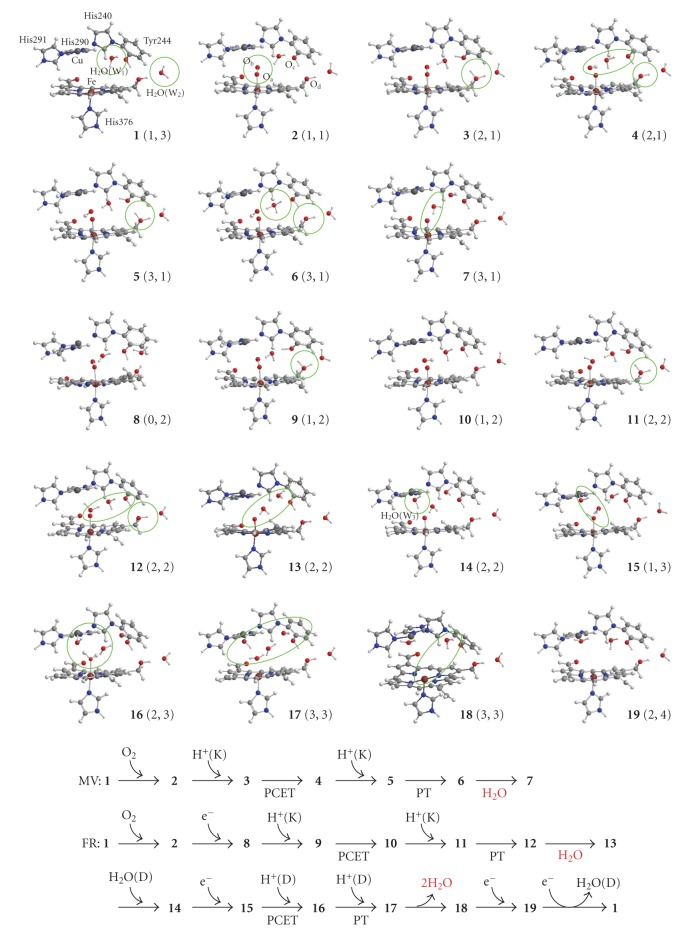
Geometries optimized on the pathways of the O_2_ reduction at the catalytic sites in MV and FR C*c*Os, and the reaction paths of MV and FR C*c*Os examined in this study.

Figure 2 shows nineteen intermediates optimized in this study. The reduction pathways of O_2_ molecule at the catalytic sites of the MV and FR C*c*Os are also shown to make easy understanding our reaction scheme estimated in this study. Their total energies, expectation values of square of spin angular momentums, and relative energies are summarized in Table 2. The Mulliken charge and spin populations of atoms and groups are summarized in Tables S1–S6 in Supplementary material available at doi: 10.1155/2010/182804. The atomic distances between key atoms are also tabulated in Table 3.

### 3.1. Early Stage of the O_2_ Reduction (1–2)

#### 3.1.1. On H_2_O Coordination to Cu in the *C* *u*_B_ Site

**Figure 3:**
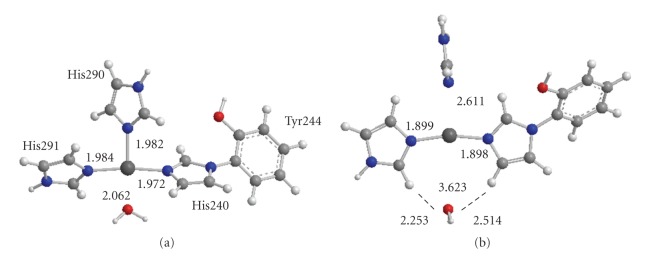
Optimized geometries of the Cu_B_ site in which the Cu atom is coordinated by a H_2_O molecule as fourth ligand. The Cu atoms are an oxidized Cu(II) (A) and a reduced Cu(I) (B).

When the catalytic site is an oxidized state [Fe(III) Cu(II)], it was shown that the H_2_O molecule coordinated to the Cu atom of the Cu_B_ site plays a crucial role for the formation of second H_2_O molecule from Fe=O of the heme *a*_3_ site [79]. However, it is not clear whether the H_2_O molecule coordinates to the Cu(I) atom in the reduced [Fe(II) Cu(I)] catalytic site at an early stage of the reduction or not. In order to account for possibility of coordination of H_2_O, the full geometry optimizations of the Cu_B_ site with and without H_2_O were carried out. The optimized geometries with and without H_2_O are shown, respectively, in Figure 3 and Supplementary Figure S1.

It is apparent from Figure 3 that the geometry of Cu(II) is different from that of Cu(I). For the oxidized Cu(II), the distances between Cu and N of His290, His291, and His240 are, respectively, 1.982, 1.984, and 1.972 Å, comparable with 1.957, 1.913, and 2.162 Å of the reduced 1OCR and 1.914, 1.920, and 2.194 Å of the oxidized 1OCC. The distance of H_2_O toward Cu atom is given by 2.062 Å, showing that the H_2_O molecule coordinates to the Cu atom as a fourth ligand. Thus, the optimized geometry is in reasonable agreement with the X-ray structures of the reduced 1OCR and oxidized 1OCC. However, the optimized geometry of the reduced Cu(I) deviates remarkably from the X-ray structures. Three histidines are rotated around the N–Cu bond. The distance between Cu and N of His290 is 2.611 Å, being remarkably longer by 0.654 Å than 1.957 Å of 1OCR. A notable distance is 3.623 Å between H_2_O and Cu, being remarkably longer than 2.062 Å of the Cu(II) geometry. However, the O atom of H_2_O has the distances of 2.514 and 2.253 Å toward the H atoms of His240 and His291, respectively, indicating that the H_2_O molecule is weakly bound to His240 and His291 by the hydrogen bonds rather than the coordination to the Cu atom. Accordingly, it is probable that the H_2_O molecule is not bound to the Cu(I) atom in the reduced catalytic site [Fe(II), Cu(I)]. On the other hand, the optimized geometries of the Cu_B_ site without H_2_O molecule are shown in Supplementary Figure S1. Both geometries of Cu(II) and Cu(I) are almost similar to those with the H_2_O molecule shown in Figure 3. It is found that the Cu_B_ sites of the reduced and oxidized C*c*Os have similar geometries to those examined here, if they do not have any constraints such as the surrounding peptide bonds and amino acid residues. Therefore, it can be considered that the Cu_B_ site in the reduced 1OCR observed by the X-ray crystallographic study is energetically activated by the steric hindrance, while the oxidized Cu_B_ site is energetically stable with release from the steric hindrance.

#### 3.1.2. FeOO in Heme *a*_3_ Site

It is reasonable to begin the examination of the O_2_-reduction path from the reduced catalytic site, [Fe(II) Cu(I)] shown in Scheme 1, since [Fe(II) Cu(I)] is a common state for both MV and FR catalytic sites. Figure 2 shows the geometry of [Fe(II) Cu(I)] (**1**) (same as** 1 **shown in Figure 1). The distances between H(W_1_) and O(Tyr244), between O(W_1_) and H(His290), and between H(–CH_2_OH) and O(W_2_) are estimated to be 1.918, 2.117, and 1.770 Å, respectively, showing that W_1_ is hydrogen-bonded to both His290 and Tyr244, and W_2_ is also hydrogen-bonded to the farnesylethyl group. It is, thus, apparent that the network of the hydrogen bonds from W_2_ to W_1_ through the farnesylethyl and Tyr244 is constructed. Since the spin density of Fe atom in heme *a*_3_ is 2.151e (Supplementary Table S1),** 1 **is a triplet spin-state and has two unpaired spins localized on the Fe atom.

Since the electronic structures of [FeOO] in the intermediate** 2 **have been well characterized [77, 78, 102–105], those are briefly commented here. The intermediate** 2 **is a singlet state where an O_2_ molecule is bound to the Fe atom of heme *a*_3_ and is 3.5 kcal/mol lower in energy than the triplet state (Table 1). It is found from spin populations shown in Supplementary Table S1 that the FeOO moiety has the antiferromagnetically coupled spins localized on the Fe atom and OO bond, consistent with the 〈*S* ^2^〉 value of 0.9297 larger than 〈*S* ^2^〉 = 0.0 of the pure singlet spin-state, as shown in (2). Two unpaired spins occupy the bonding and antibonding orbitals of 3d on Fe and π* on OO, such as 3d_yz_ + *π*_y_* and 3d_yz_ − *π*_y_*. The spin population of the Fe atom is 1.062e, indicating that the Fe atom is oxidized from Fe(II) of** 1 **to Fe(III) with one electron transfer from the Fe atom to the OO bond. Therefore, at this stage of the reduction, the OO bond receives one electron necessary to reduce the OO bond from the reduced Fe atom.

### 3.2. Reduction Mechanism of MV CcO (2–7 in Figure 2)

It was shown in the previous work [80] that the cleavage of the OO bond occurs when FeOO on porphyrin ring receives two electrons and two protons. The OO moiety in FeOO receives already one electron from the Fe atom to give the electronic structure of Fe(III)-OO^−^. Accordingly, in the case of MV C*c*O, the OO moiety has to receive sequentially one electron from the Cu atom of the Cu_B_ site and two protons from the outside of the catalytic site.

It is reasonable to suppose that the proton transfers to OO to yield FeOOH through the hydrogen-bond network from W_2_ to W_1_, since W_2_ hydrogen-bonds to the terminal Thr316 residue of the K-pathway. The intermediate **3** shown in Figure 2 corresponds to the geometry where a proton from the K-pathway is trapped on –CH_2_OH. It can be easily seen from Tables S1 that the electronic structures of Cu and FeOO portions do not change from those of the unprotonated state **2**. Interestingly, it is found from Table 3 that W_1_ approaches to the proximal O_b_ atom of FeOO with shortening the distance of O_b_-O(W_1_) from 3.652 to 2.801 Å. The distance between the O_c_ atom of phenol and W_1_ is also made shorter from 2.871 to 2.561 Å, indicating that the hydrogen bond of phenol and W_1_ is made stronger. Thus, it is apparent that the addition of the proton to the catalytic site from the K-pathway induces formation of the stronger network of the hydrogen bonds in order to open a pathway of the proton transfer from –CH_2_OH to FeOO through Tyr244 and W_1_.

In the intermediate **4 **shown in Figure 2, FeOOH is formed. In the change from **3** to **4**, protons move simultaneously from –CH_2_OH to Tyr244, from Tyr244 to W_1_, and from W_1_ to FeOO. From Table 2,** 4 **is 33.5 kcal/mole more stable than **3**. From Supplementary Table S1, the protonated FeOOH has negative charge of −0.561e not nearly equal to zero, similar to −0.599e of the OO moiety in **3**. The spin population of OOH disappears to 0.071e from −0.959e of OO in 3, while the spin population of the Cu atom grows up from −0.039e to −0.507e. In the formation of** 4 **from **3**, the antiferromagnetic spin coupling shifts from between Fe and OO in **3** to between Fe and Cu in **4**. These indicate that one electron transfers from the Cu atom to the OOH moiety with changing the oxidation state of the Cu atom from Cu(I) to Cu(II), consistent with the increase of the bond distance of OO from 1.307 Å to 1.449 Å. It should be noted here that at this stage of the reaction the OOH moiety receives two electrons from the reduced Fe and Cu atoms and one proton from the K-pathway.

In order to explore the formation of** 4 **from **3** in more details, the H-atom on –CH_2_OH_2_^+^ moved toward the O_c_-atom of Tyr244 in a stepwise manner. Supplementary Figure S2 shows the change of the relative energy, the variations of charge and spin populations for the key atoms and OO (OOH) moiety and the atomic distances. The relative energy rapidly decreases from 7.4 to −14.4 kcal/mol in range of 1.4 and 1.35 Å. Supplementary Figure S3 shows the geometries **3a** and **4a** at R_OH_ = 1.4 and 1.35 Å, respectively. It is found that the proton of Tyr244 transfers simultaneously to W_1_ in the geometry** 3a **with the proton transfer from −CH_2_OH to Tyr244. At 1.35 Å, the proton of Tyr244 has transferred to W_1_, and simultaneously the other proton of W_1_ has transferred to FeOO to yield the FeOOH moiety. From simple insight, it seems that W_1_H^+^, which is formed by receiving a proton from Tyr244, blows off the other proton to FeOO. However, W_1_H^+^ is a transient state on the potential energy surface [78]. With decreasing the energy without barrier, W_1_H^+^ moves to approach to proximal O_b_ of FeOO, and at about 2.6 Å a proton shifts from W_1_H^+^ to FeOO to give FeOOH, and remainder W_1_ switches back to the original position to give the state **4a**. It can be seen from the change of the spin populations that the electron transfers from Cu to FeOOH at the same time of formation of FeOOH. Apparently the structural change from 1.4 to 1.35 Å is continuous. Accordingly, the reaction from **3** to** 4 **proceeds in mechanism of the proton-coupled electron transfer (PCET) with the activation energy of about 7.4 kcal/mol.

The intermediate **5** corresponds to the geometry that a proton from the K-pathway is captured on –CH_2_OH of farnesylethyl group in **4**. **5** is a singlet spin-state that the antiferromagnetic spin coupling exists on Fe(III) and Cu(II). Similar to the formation of FeOOH from** 3** to **4**, the H-atom on –CH_2_OH_2_ **^+^** moved toward O_c_ (Tyr244) from the geometry of **5**. A proton of Tyr244 moves simultaneously to W_1_. The formed W_1_H^+^ does not move toward FeOOH to yield FeOOH_2_ or FeO + H_2_O, in contrast to the case from** 3** to **4**. The geometry **6** with W_1_H^+^ was obtained. The variations of energy and spin populations with moving the proton to O_c_ of Tyr244 is shown in Supplementary Figure S4. The activation energy of the proton transfer is about 9.3 kcal/mol, slightly higher by 1.9 kcal/mol than that from** 3** to **4**. The geometry **6** is 5.4 kcal/mol lower than **5**, smaller than 33.5 kcal/mol from** 3** to **4**. From Supplementary Table S2, the change from **5** to **6** proceeds in the proton transfer without the electron transfer from the Cu(II) atom.

The geometry **7** corresponds to the intermediate where the H_2_O molecule is formed by cleaving the OO bond and moving the proton from W_1_H^+^ to separated OH^−^. The **7** is 15.4 kcal/mol lower than **6**, and is a singlet spin-state with the expectation value of the squared spin angular momentum of 2.0580, indicating existence of two pairs of the antiferromagnetic spin couplings in **7**. The spin population of Fe=O is given by 2.078e with two parallel unpaired spins distributed over Fe=O, showing Fe(IV)=O^2−^. The molecular orbitals corresponding to Fe=O are composed of two antibonding orbitals of d_yz_ (Fe)-p_y_ (O) and d_xz_ (Fe)-p_x_ (O), which are the same as those of the naked heme(Fe)=O [78, 80]. The Cu atom has the spin population of −0.590e (−1.001e for the Cu_B_ site), showing that the oxidation state of the Cu atom does not change from Cu(II), compared with those of **6**. However, the spin population of the porphyrin ring decreases in negative value from −0.350 to −1.089e, and the charge population increases from −0.729 to −0.173e. This indicates that the porphyrin ring loses one electron and has single unpaired electron of the antiparallel spin to the Fe=O. Accordingly the heme *a*_3_ site is thought to be the compound I with the radical cation of the porphyrin ring [78, 80, 106–108], consistent with the experimental results of the time resolved Raman spectroscopy [109, 110]. The Fe–O_a_ distance is estimated to be 1.658 Å, comparable with 1.64–1.70 Å determined by experiments [111, 112], and with 1.669 Å of theoretical value [106].

In order to confirm the connection from the state **6** to **7**, the O–O distance of FeOOH is increased. Supplementary Figure S5 shows the energy change with increasing the O–O distance from the state **6**. The energy increases and has a maximum of 4.9 kcal/mol at *R*_OO_ = 1.8 Å. Supplementary Figure S6 shows the geometries at *R*_OO_ = 1.6 and 1.7 Å. It is easily found that the proton moves from W_1_H^+^ to FeOOH^−^ to yield the H_2_O molecule at the early stage of the OO-bond cleavage. After passing *R*_OO_ = 1.8 Å, the energy decreases gradually and crosses to the potential energy surface connecting to the state **7.**

For MV C*c*O, only one water molecule was produced by two-electron reduction of the oxygen molecule. Two electrons are provided from the reduced Fe(II) of heme *a*_3_ and Cu(I) of the Cu_B_ site, while two protons are provided from the network of hydrogen bonds including W_1_, Tyr244, –CH_2_OH, and W_2_ connecting to the terminal Thr316 of the K-pathway. When Fe(III)OOH^−^ (4) is formed from Fe(III)OO^−^ (2) by the proton transfer, the electron transfers from Cu(I) to Fe(III)OO^−^ in manner of PCET. On the pathway from **6** to **7** where the H_2_O molecule is produced, the recombination of the electronic structure occurs at the catalytic site, in good agreement with observation that the reduction in MV C*c*O is 5-6 times slower than in FR C*c*O [2, 3, 32].

### 3.3. Reduction Mechanism of FR CcO

#### 3.3.1. First H_2_O Formation (2, 8–13 in Figure 2)

On the contrary to MV C*c*O, in FR C*c*O, there are two more electrons to reduce the O_2_ molecule in the reaction system, heme *a* and Cu_A_ site. It is, thus, expected that one electron is put into the catalytic site from heme *a* after the intermediate** 2 **is formed. The geometry **8** in Figure 2 is a one-electron reduced state of **2**.

The 8 is 81.0 kcal/mol lower than **2**, indicating the possibility that the O_2_-adduct** 2 **to heme *a*_3_ can receive easily an electron. **8** is also the bound state with 60.5 kcal/mol lower than the dissociation state of** 1 **and O_2_^−^. It is found from Supplementary Table S3 that the unpaired spin is localized on the OO moiety with small distribution of 0.157e on the Fe atom, showing that **8** is reduced by the addition of one electron with changing the oxidation state from Fe(III) to Fe(II). The charge populations on the porphyrin ring and OO moiety are increased in negative values, showing that the paired electrons are delocalized to the porphyrin ring and OO moiety. These features are consistent with the results for the reduced heme [113, 114].

It is reasonable to consider that the increases of the electron-negative characteristic on the porphyrin ring and OO moiety enhance the possibility of receipt of a proton. It is expected that the proton is provided to the catalytic site through the K pathway similar to the MV C*c*O. The intermediate **9** is an optimized geometry where the proton is trapped on –CH_2_OH with decreasing the energy by 0.394 au from **8**, compared with 0.299 au from **2** to **3**. It is found from Table 3 that W_1_ approaches to the proximal O_b_ atom of FeOO with shortening the distance of O_b_-O(W_1_) from 3.073 to 2.593 Å. The distance of O_c_-O(W_1_) is also shortened from 2.891 to 2.481 Å. These show that the hydrogen bonds are made stronger among phenol, W_1_, and O_b_ of FeOO.

Similar to the variation from **3** to** 4 **in MV C*c*O, the H atom of Tyr244 was moved toward O of W_1_ from b. It is found from Supplementary Figure S7 that the relative energy is rapidly decreased in the region from 1.3 Å to 1.2 Å, being similar behavior to the rapid decrease from 1.4 Å to 1.3 Å to yield FeOOH (4) from FeOO (3) in the MV C*c*O. In this region, the H atom of W_1_ transfers as a proton to FeOO (9) to give FeOOH (10). This structural change is fairly similar to that found in MV C*c*O. The spin populations of Fe and OO are rapidly increased from 0.3 to 0.95e and decreased from 0.9 to 0.1e, respectively. On the contrary, the oxidation state of the Cu atom maintains Cu(I). This shows that an electron of Fe transfers to the OO moiety to make a paired spin with an unpaired electron of OO. Namely, the proton transfer occurs concertedly with the electron transfer from Fe to OO, being different from the electron transfer from Cu to OO in MV C*c*O. From the small activation energy, the proton from K-pathway transfers to Tyr244 without the capture on –CH_2_OH. The formed** 10 **is 55.3 kcal/mole lower than **9** and has an unpaired spin localized on the Fe atom in **10**. At this stage of the reaction, the OOH moiety receives two electrons and one proton necessary for performing the reduction of the O_2_ molecule. (Supplementary Figure S7).

**Scheme 2:**
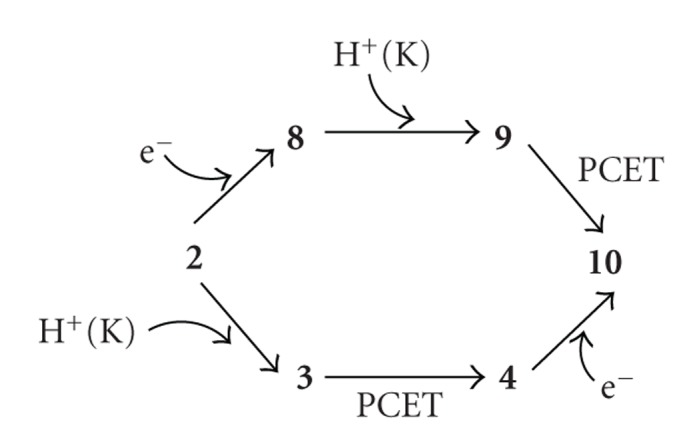
Two reaction pathways to produce FeOOH (**10**) from FeOO (**2**).

In order to yield the FeOOH (**10**) from FeOO (2), the electron and proton were sequentially added. However, the alternative path to obtain** 10 **can be considered, as shown in Scheme 2. The path from** 2 **to** 10 **through **8** and **9** has been mentioned in this section. The path from** 2 **to** 4 **through** 3** was also mentioned as a path of the MV C*c*O in the preceding section. The catalytic site of** 4 **is simply presented by [Fe(III)-OOH^−^ Cu(II)]. The [Fe(III)-OOH^−^ Cu(I)] (**10**) can be easily obtained by addition of an electron to the catalytic site of** 4 **from the heme *a* site, if the added electron occupies the 3d orbital of the Cu atom. Actually, in our calculation, the addition of an electron to** 4 **gave** 10 **with decrease of the energy by 0.2588 au, as found from Table 2. We would like to discuss later which path is favorable.

The geometry** 11 **is an intermediate where a proton from the K-pathway is trapped on –CH_2_OH, corresponding to **5** in formation from FeOOH to FeO + H_2_O. Similar to the proton transfer from** 3** to **4**, the H-atom on –CH_2_OH_2_^+^ was shifted toward O(Tyr244) from the geometry of 11. Supplementary Figure S8 shows the change of the relative energy. In contrast with the rapid decrease of energy from** 3** to **4**, the change of energy shows the smooth curve to connect continuously to the state** 12 **with the activation energy of about 6.4 kcal/mol. The intermediate** 12 **is 14.6 kcal/mol lower than **11**, which is remarkably smaller than 33.5 kcal/mol from **3** to **4**. (Supplementary Figure S8)

The** 12 **has the structure of FeOOH_2_ where H is added to FeOOH of **11**. The charge population of the porphyrin ring and OOH moiety changes from −0.413 to −1.032e and from −0.583 to −0.065e, respectively. The spin populations of the porphyrin ring and OOH moiety do no change. This shows that the added H to FeOOH is a proton without any electron transfer. The OO scission does not occur with the OO distance of 1.484 Å which is slightly longer than 1.453 Å in the state **11**, in contrast with the OO-bond cleavage on the naked heme by receiving two electrons and two protons [80]. This might be due to the hydrogen bond to W_1_ which is hydrogen-bonded to His290 and Tyr244.

We cleave the OO bond from the geometry of **12**. Supplementary Figure S9 shows the changes of the relative energy. The energy gradually increases with breaking the OO bond and decreases through the maximum point at *R*_OO_ = 1.9 Å. On the optimization at *R*_OO_ = 2.0 Å using the geometry optimized at *R*_OO_ = 1.9 Å, the energy was rapidly decreased. As can be seen in Supplementary Figure S10, the difference in two geometries at *R*_OO_ = 1.9 and 2.0 Å is found in directions of OH bond in OOH_2_. The OH of H_2_O faces to the Cu_B_ site at *R*_OO_ = 1.9 Å, while the OH faces to the porphyrin ring at *R*_OO_ = 2.0 Å. Using the optimized geometry and the molecular orbital at *R*_OO_ = 2.0 Å, we carried out again the geometry optimization at *R*_OO_ = 1.8 Å. The geometry where the OH faces to the porphyrin ring was obtained. It possesses 5.1 kcal/mol lower in energy than the original geometry where OH faces to the Cu_B_ site. Decreasing the OO distance from geometry at *R*_OO_ = 1.8 Å, the potential energy curve crosses with the original curve at *R*_OO_ = 1.7 Å and has a maximum at *R*_OO_ = 1.65 Å. Finally the minimum energy point, **12**a, was obtained with the OO distance of 1.489 Å, comparable with 1.484 Å of **12**. The** 12a **is only 2.4 kcal/mol higher than **12**, showing that** 12a **has higher possibility to cleave the OO bond because of lower activation energy of 2.3 kcal/mol than 7.6 kcal/mol from **12**. From Tables S4, the charge and spin populations of** 12a **are the same as those of 12. The change of direction of the OH bond from** 12 **to** 12a **has small activation energy of 3 kcal/mol, showing the possibility to easily convert from** 12 **to** 12a **before the OO bond breaking.

On the other hand, the increase of the OO bond length leads to the monotonous decrease of the total energy and finally the intermediate** 13 **was obtained as a minimum geometry. The** 13 **is 25.1 kcal/mol lower in energy than **12**a. Interestingly, it is apparent from the geometry shown in Figure 2 that the first H_2_O molecule is formed with small activation energy of 2.3 kcal/mol. The spin populations of the FeO moiety formed are 2.081e (= 1.304e(Fe) + 0.774e(O)), showing that the Fe=O moiety has two unpaired spins with parallel direction. The spin populations of the Cu_B_ site grow up from zero value to −0.711e in negative value. The spins of Fe (*S* = 1) and Cu (*S* = 1/2) are antiferromagnetically coupled, in agreement with experimental proposal [32]. This shows that the oxidation state of the Cu atom alters from Cu(I) to Cu(II) with loss of one electron, consistent with change of the charge population of the Cu_B_ site from 1.095e to 1.536e. Therefore, it is formally considered that one electron of Fe(III) transfers to the O atom and one electron of Cu(I) also transfers to the O atom to yield the Fe(IV)=O^2−^ bond. Accordingly, the heme *a*_3_ of** 13 **is a compound II, even that the porphyrin ring has small spin population.

**Figure 4:**
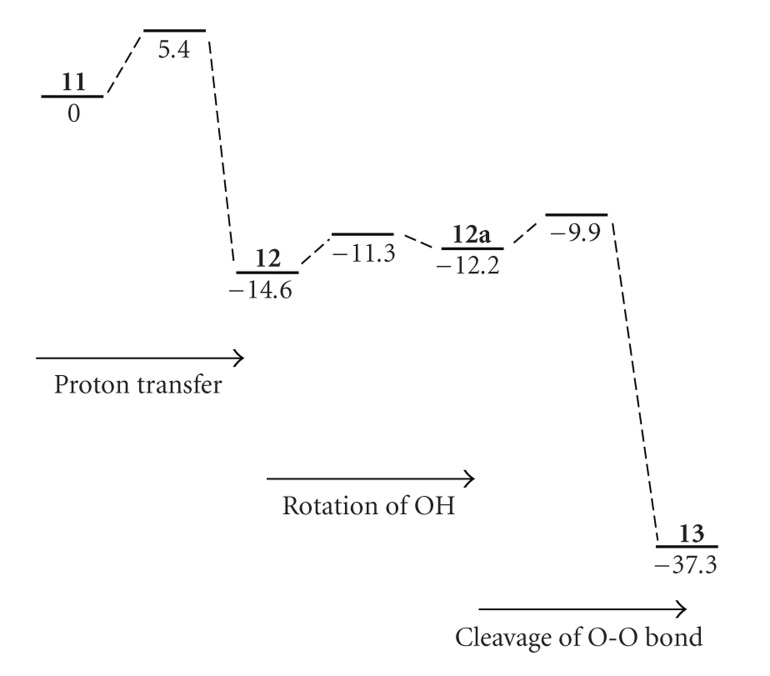
Energy diagrams for formation of first H_2_O molecule in FR C*c*O.

Figure 4 summarizes schematically the energy variations for the formation of first H_2_O molecule from** 11 **to **13**. At the early stage of the reaction from **11**, the proton transfers to FeOOH to yield** 12 **(FeOOH_2_) through the K-pathway with the activation energy of 5.4 kcal/mol and exothermic energy of 14.6 kcal/mol. After** 12 **was formed,** 12 **is converted to** 12a **with the rotation of OH in FeOOH_2_ in order to connect smoothly to **13**. The rotation barrier is estimated to be 3.3 kcal/mol, showing that the transition state is extremely lower than **11**. Consecutively, the OO bond cleavage is induced to produce first H_2_O molecule with small activation energy of 2.3 kcal/mole and exothermic energy of 25.1 kcal/mol. The rate determining step of the reaction from** 11 **to** 13 **is the first one from** 11 **to **12**. Since **12**, **12**a, and** 13 **are lower in energy than **11**, this reaction easily proceeds when the catalytic site captures one proton from the K-pathway. It is probably considered that the rotation of OH and cleavage of the OO bond occurs concertedly without forming** 12 **and** 12a **to yield the desired H_2_O molecule.

#### 3.3.2. Second H_2_O Formation (14–17 in Figure 2)

As mentioned above, three electrons from heme *a*, Cu, and Fe and two protons from the K-pathway have been used to produce a first H_2_O molecule. Thus, second H_2_O molecule should be produced by remainder one electron and two protons. It has been shown in a recent study [57, 58] that the D-pathway links to the catalytic site through the hydrogen-bond network of water molecules. The oxidation state of Cu in** 13 **is an oxidized Cu(II). As discussed in Section 3.1.1, the oxidized Cu(II) has possibility of fourth ligand of the H_2_O molecule. Thus it is reasonable to consider that the D-pathway is open for the hydrogen-bond pathway connecting to the Cu atom in the Cu_B_ site to make coordination of H_2_O to Cu after the intermediate** 13 **was formed.

The structure where the H_2_O molecule (W_3_) coordinates to the Cu atom is shown as** 14 **in Figure 2. From charge and spin populations shown in Supplementary Table S5, it is found that the electronic structure of** 14 **is similar to that of **13**, even though the spin population on the Cu atom is slightly enhanced. The distance between Cu and O of the coordinating H_2_O (W_3_) is estimated to be 2.037 Å, in good agreement with 2.062 Å of the Cu_B_ site model in Figure 3. The distance between H of W_3_ and O_a_ of Fe=O_a_ in heme *a*_3_ is estimated to be 1.592 Å, longer than 1.435 Å given in the previous work [79]. However, 1.592 Å is slightly shorter than the standard hydrogen-bond distance. Thus, the added W_3_ coordinates to Cu in the Cu_B_ site and makes the hydrogen bonding to Fe=O in heme *a*_3_, simultaneously. The Fe=O distance of 1.676 Å is unchanged from 1.660 Å in** 13 **upon the addition of H_2_O molecule.

The last one electron of four electrons necessary for the reduction of O_2_ was added to** 14 **without changing the geometry of **14**. The subsequent geometry optimization induces the proton transfer from W_3_ on Cu to O_a_ of Fe=O_a_, giving** 15 **with FeOH in heme *a*_3_. Obviously, the one-electron reduced **14** ^−^ is a transient state on the potential energy surface. The Fe–O and O–H distances of FeOH are given to be 1.841 Å and 0.991 Å, respectively, showing formation of a strong OH bond on Fe. The 〈*S* ^2^〉 value of** 15 **is found to be 2.0192, being close to 2.0 of the pure triplet spin-state. The spin population of Fe plus OH(Fe) is 1.047e, while that of the Cu_B_ site (Cu_B_ plus OH(W_3_)) is 1.001e, indicating that two up-spins are localized on Fe and Cu, respectively. Thus, the oxidation state of the Fe atom changes from Fe(IV) of** 14 **to Fe(III) with keeping the oxidation state of Cu(II). However, the spin population on Cu is discontinuous because of the change from negative value of** 14 **to positive one of 15, implying the spin-flip on Cu from** 14 **to 15. In the **14** ^−^ state, the added electron occupies 3d orbital of the Cu atom to change the oxidation state from Cu(II) to Cu(I), while the Fe=O moiety keeps two parallel up-spins. With the proton transfer from W_3_, an electron of the down-spin in the Cu atom simultaneously transfers to Fe=O, yielding the Fe(III)-OH^−^ and Cu(II)-OH^−^ of **15**. This concerted proton-electron transfer gives the continuous change from the 14^−^ state to **15**. 

Two protons remain to produce a second H_2_O molecule from the intermediate **15**. One proton was added to OH^−^ on Cu in** 15 **under the assumption that the proton enters the catalytic site through the D-pathway. This 15H^+^ is also a transient state on the potential energy surface. The geometry optimization leads to the proton transfer from the formed H_2_O (W_3_) to FeOH, yielding the H_2_O molecule on the Fe atom as a second productive H_2_O, as shown in **16**. Compared the charge and the spin populations of Cu and Fe in** 15 **and **16**, those stay invariant through the proton transfer, showing that the oxidation states of Cu and Fe remain unchanged from Cu(II) and Fe(III). Accordingly, these features show that the change from** 15 **to** 16 **is a simple proton transfer without electron transfer, being different from the concerted proton-electron transfer from **14** ^−^ to **15**. In the structure of **16**, the distance of Fe and the formed H_2_O is given by 1.928 Å, showing that the formed H_2_O is weakly bound to heme *a*_3_. This is due to the strong attraction of the hydrogen bond to OH^−^ coordinating to the Cu atom.

At this stage of the reaction, the aimed second H_2_O molecule has been produced, although the fourth proton still remains unused for the reduction in the catalytic site. It is reasonable to consider that the fourth proton enters to neutralize the OH^−^ on Cu through the D-pathway. The neutralized geometry is shown as **17**. The second H_2_O molecule is slightly separated from Fe with changing the distance from 1.928 Å in** 16 **to 2.057 Å in **17**. The Fe and Cu atoms are oxidized with the oxidation states of Fe(III) and Cu(II), compared with reduced states of Fe(II) and Cu(I) at the starting point of **1**. The state** 17 **is a triplet spin-state with the ferromagnetic coupling of two unpaired spins on Fe and Cu. At this stage of the reaction, the O_2_ molecule is reduced to two H_2_O molecules by four electrons and four protons.

#### 3.3.3. Catalytic Cycle (17–19, 1)

Both the Fe and Cu atoms should be reduced to complete the catalytic cycle, since the Fe and Cu atom in** 17 **are oxidized. At this stage of the reaction, the formed two water molecules will be excluded from the catalytic site. The geometry without two H_2_O molecules is shown as** 18 **in Figure 2. The Cu-O(W_3_) distance does not alter from 2.068 Å of** 17 **to 2.042 Å of **18**. After exclusion of two water molecules, it is expected that two electrons are sequentially put into the catalytic site from heme *a* in order to reduce both Fe and Cu atoms. The first electron occupies the 3d_*z*^2^_ orbital of the Fe atom not the 3d_yz_ orbital, giving the intermediate** 19 **with three parallel spins localized on Fe and Cu.** 19 **is a quartet spin-state.

The second electron occupies 3d orbital to reduce the Cu atom and simultaneously the W_3_ coordinated to the Cu atom is released because of Cu(I), leading to the closure of the D-pathway. Finally, the reduced catalytic site of** 1 **reverts to perform the next reduction of the O_2_ molecule. At this stage, the catalytic cycle of FR C*c*O is completed.

## 4. Summary of Reduction Mechanism

As shown in Scheme 1, several intermediates have been experimentally observed in the reduction of O_2_ molecule. Their intermediates have been assigned by R, A, and P_M_ in reduction by MV C*c*O, while R, A, P_R_, F, and O have been assigned in this order through the catalytic reaction by FR C*c*O. There is consensus that the intermediate R is composed of the reduced Fe(II) and Cu(I) and A has the structure where the O_2_ molecule is bound to Fe in heme *a*_3_. However, the proposals for structures of P_R_, F, and O are in debate.

**Figure 5:**
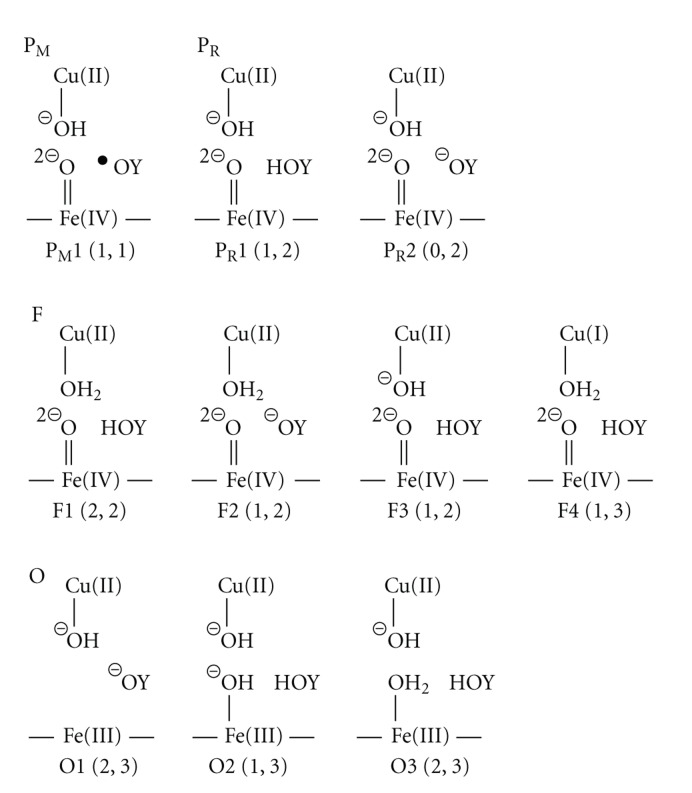
Schematic structures of the catalytic sites of P_M_, P_R_, F, and O proposed in experimental studies.

Summarized in Figure 5 are the schematic structures which have been proposed by experimental studies up to now. HOY shows a neutralized Tyr244, while ^−^OY and ^*·*^OY shows a deprotonated Tyr244 and a neutralized Tyr244 radical, respectively. Also shown in the parenthesis are total charge and spin multiplicity that are estimated from the proposed structure. For each of P_R_, and F, those are apparently conflicting in the state of the Cu_B_ site including Tyr244. These might be from speculation due to the fact that the Cu_B_ site is silent for observations of EPR and spectroscopy and the phenol has properties of a proton and electron donors. Several points are, however, common for P_M_, P_R_, and F. The heme *a*_3_ moiety has the electronic structure of Fe(IV)=O^2−^ and compound II where the porphyrin ring is neutral. In the bond of Fe(IV)=O^2−^, two spins are coupled ferromagnetically.

**Scheme 3:**
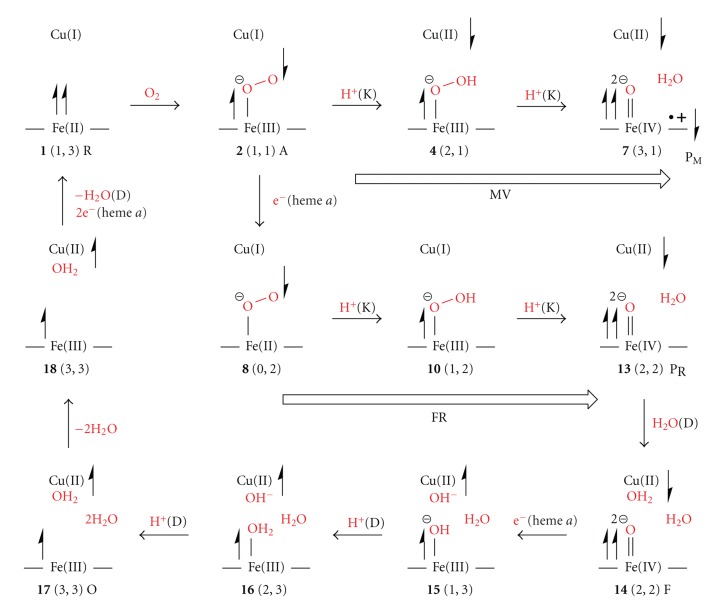
Schematic representation of the mechanisms for O_2_-reduction by MV and FR C*c*Os.

In this work, we theoretically examined the reduction mechanisms of O_2_ molecule at the catalytic sites of MV and FR C*c*Os. Our mechanisms are summarized in Scheme 3. The intermediate A (2) is produced by binding O_2_ on Fe of heme *a*_3_ in the reduced state R (1). The reduced Fe atom is oxidized and an electron of Fe transfers to dioxygen, yielding Fe(III)-OO^−^ in the singlet biradical state.

For MV C*c*O, after A is formed, two protons are sequentially added to the catalytic site through the K-pathway. The first proton transfer provides the Fe(III)OOH^−^ (4) with simultaneous electron transfer from Cu(I) to FeOOH. The activation energy for the proton transfer was estimated to be 7.4 kcal/mol. The addition of the second proton leads to the OO bond cleavage to produce the H_2_O molecule (**7**). As shown in Supplementary Figure S5, the recombination of the electronic structure occurs at the catalytic site, in good agreement with the observation that the reduction in MV C*c*O is 5-6 times slower than in FR C*c*O [2, 3, 32]. The heme *a*_3_ is a compound I with a radical cation of the porphyrin ring, consistent with the experimental result of the time resolved Raman spectroscopy [109, 110].

We assign the intermediate **7** as P_M_, being in conflict with P_M_1 [22, 32, 43, 44, 74, 83, 115] shown in Figure 5. The intermediate P_M_1 is obtained under the consideration that the proton transfers from Tyr244 to FeOO to yield hydroperoxide and subsequently one electron transfer from Cu_B_ is induced to cleave O–O bond [3]. Then third electron transfers from Tyr244 to Fe-O to yield Fe(IV)=O^2−^ and tyrosyl radical. As shown in **4**, on the pathway that the Fe(III)OOH^−^ is formed, an electron certainly transfers from Cu(I) to FeOOH. However, the cleavage of the OO bond does not occur in** 4**. The OO bond breaking necessitates the addition of one proton to Fe(III)OOH^−^. In our trial calculations (not shown here), P_M_1 is 12.4 kcal/mol higher than A, indicating that the reaction from A to P_M_1 is endothermic. The distance between H of phenol and O_b_ of FeOO is estimated to be 4.697 Å, which is too far to perform the proton transfer. The reasonable distances for the proton transfer are in the range of 1.4–1.8 Å. At least one more H_2_O molecule is necessary to induce the proton transfer between FeOO and Tyr244 [70, 71]. Even the H_2_O molecule(s) are added, the endothermicity of the reaction will not be changed. In addition, the phenoxyl radical has *π*-character, not *σ*-character. The pathway interacting with the *π*-orbital of the phenoxyl radical is necessary to induce the smooth electron transfer from Tyr244 to FeO. However, it is not expected from the structure of the catalytic site.

It can be thought in our examinations that the intermediate A is a branching point to divide mechanisms of MV and FR. For FR C*c*O, before two protons transfer from the K-pathway, an electron transfers from heme *a* to the catalytic site with changing the oxidation state of the Fe atom from Fe(III) to Fe(II). After the state **8** was formed, two protons sequentially transfer from the K-pathway to reduce the dioxygen of FeOO. As discussed in Scheme 2, there is an alternative pathway that the order of the electron and proton transfer is reversed. The addition of an electron to** 4 **leads to **10**. The path from** 2 **to** 10 **through** 4 **has the proton acceptability (proton affinity) of 0.2989 au (Table 2) from the K-pathway and the activation energy of 7.4 kcal/mol for the proton transfer in the catalytic site. The addition of an electron to the intermediate A to provide the state **8** induces decreasing the energy by 0.1291 au (positive electron affinity). The state **8** has the higher proton acceptability of 0.3938 than 0.2989 au from A to **3**. There is no activation energy (~0.2 kcal/mol) for the proton transfer to produce the state **10**. Thus, the path A–**8**–**10** is preferable to the path A–**4**–**10**, in agreement with the experimental proposal that an electron transfers from heme *a* to heme *a*_3_ after the intermediate A is formed [61, 64].

We assign the intermediate** 13 **as P_R_, being in conflict with P_R_1 [32, 43, 74] and P_R_2 [22, 44, 56, 83, 115] except for Fe(IV)=O^2−^ in compound II. In the Cu_B_ site, the** 13 **has the oxidized Cu(II) without any ligand, while both P_R_1 and P_R_2 have a hydroxy anion. P_R_2 has the ^−^OY anion at the site of Tyr244 and is connected by addition of one electron to P_M_1. As mentioned above, these conflictions are originated by differences of the procedure of the proton donation to the dioxygen. HOY plays roles of the donations of a proton and an electron to FeOO in P_R_1 and P_R_2, while it aids to transfer a proton from the K-pathway without any electron transfer in our P_R_. When **8** changes to **9** in which the proton was trapped on –CH_2_OH of heme *a*_3_, W_1_ approaches to both FeOO and HOY to make the strong network of the hydrogen bonds from the K-pathway to FeOO through W_2_, –CH_2_OH, HOY, and W_1_. Thus, W_1_ moves to help the transportation of the proton from the K-pathway to the dioxygen moiety. In the process of formation of first H_2_O molecule (**2**, **8**–**13**), since W_1_ walks around the space composed of FeOO, HOY, and His290, W_1_ will not be detectable in the X-ray crystallographic measurement [7–12]. It is thought that the first H_2_O molecule cannot be formed without W_1_, being consistent with the observation that the reduction does not proceed by the mutation of His290 [51–54]. Therefore, W_1_ plays a crucial role for the formation of the first H_2_O molecule in the reduction of O_2_ molecule, while HOY plays a role of a relay point for the proton transfer from the K-pathway to the dioxygen.

The oxidation state of the Cu atom maintains the reduced Cu(I) on the path from the intermediate A to P_R_, and Cu is oxidized to Cu(II) at the formation of the intermediate P_R_. During this process, the Fe atom receives an electron from heme *a* and delivers the electron to the OO moiety. The intermediate P_R_ is stabilized by the release of the energy of the steric hindrance with the change of the oxidation state of the Cu atom from Cu(I) to Cu(II), as can be seen from Supplementary Figure S1. Then the Cu atom has high potentiality of the coordination of the H_2_O molecule, as shown in Figure 3. At this stage, the D-pathway is open. An H_2_O molecule coordinates to the Cu atom, giving **14**. We assign the intermediate** 14 **as F.

For F, F1 [32], F2 [22], F3 [19, 44, 83], and F4 [43, 74] are proposed based on the spectroscopic observation. These proposed four structures have a common Fe(IV)=O^2−^, being coincident with our F. The Cu_B_ and tyrosine sites are, however, different. F4 is a transient state that is on the path from** 14 **to** 15 **in our reduction process. When the Cu atom is reduced, immediately the proton transfers from the coordinating H_2_O to FeO with the simultaneous electron transfer from Cu(I) to FeO, giving **15**. It is surprising that F3 is similar to P_R_1. We could not make a comment which is assigned to P_R_ or F. F2 is obtained by the addition of the proton to P_R_2 [22]. Our F has a neutralized HOY with the total charge of two, which is different from ^−^OY of F2. F1 coincides with our F. We would like to consider that this is an accidental agreement, because F1 is thought to be obtained by addition of the proton to P_R_1. However, it might be concluded that the intermediate F has the structure such as F1 and our F. Our assigned F has the same oxidation state as P_R_, in good agreement with the proposal that the P_R_→ F transition is not coupled with the electron transfer [61].

Sequential additions of one electron and two protons lead to the intermediate** 17 **through** 15 **and **16**. Interestingly,** 15 **is the same as O2 shown in Figure 5 [44, 115]. Also** 16 **is the same as O3 [43, 74, 83]. Similar to the sequential addition of protons in the process from **8** to **10**,** 15 **and** 16 **will not be detectable. Since it could be considered that** 17 **is stabilized, we assign** 17 **as O. For the process of the formation of the second H_2_O molecule (**14**–**17**), the Cu atom is maintained the oxidized Cu(II), in interest contrast with the process of the formation of the first H_2_O molecule (**8**–**13**) that the Cu atom is maintained the reduced Cu(I). Further, through the formation of two water molecules, the Fe atom is always the oxidized Fe(III) except for the intermediate **8** of Fe(II), and the intermediates** 13 **and** 14 **of Fe(IV)=O^2−^. The** 18 **is obtained by removing two produced H_2_O molecules from** 17 **to the outside of the catalytic site. However, the possibility that** 18 **is assigned as O is left. 

Four electrons and four protons are used to produce two water molecules for the reduction of the oxygen molecule in the catalytic site of FR C*c*O. Two of four protons are provided from the K-pathway to produce the first water molecule, while the remainder two protons are from the D-pathway to produce the second water molecule. In our reduction mechanism, the K-pathway is ahead of the D-pathway, in conflict with the reversed order proposed from the experiments [44, 60, 61]. Our mechanism is, however, consistent with the recent observation that mutations in the K-pathway slowed down the formation of the P_R_ intermediate [64].

## 5. Concluding Remarks

We have examined systematically the reduction mechanisms of the oxygen molecule in the mixed-valence and fully reduced C*c*Os and shown consistently the catalytic cycle based on the theoretical calculations. The W_1_ added to the catalytic site plays crucial roles for the production of the first water molecule. However, W_1_ is not observable for the X-ray crystallographic measurement due to the rapid motion in the catalytic site. The W_3_ coordinated to Cu also plays crucial roles for the production of the second water molecule. The Cu atom is an electron storage during the formation of the first water molecule, while the Cu atom keeps the oxidized state of Cu(II) during the formation of the second water molecule. Some aspects of our mechanism are in good agreement with the experimental proposals, but some aspects are in disagreement. In our mechanism, Tyr244 plays a relay for the proton transfer from the K-pathway to the dioxygen moiety. It is unreasonable that Tyr244 is the proton and electron donors, since the distance between Tyr244 and the dioxygen moiety is too long. The K-pathway functions for the formation of the first water molecule, while the D-pathway functions for the second molecule. This order is reversed in the experimental proposal.

We have examined the bovine C*c*O which belongs to the A1 family *a* *a*_3_ [17, 18]. The A1 family has the K- and D-pathways for the proton channels, while the B family *b* *a*_3_ has only the K-pathway. Actually, in our trial calculation for 1XME of the B family (not shown here), the proton does not easily transfer from H_2_O of the Cu_B_ site to Fe=O on the way from** 14 **to** 15 **[74]. The reduced **14** ^−^ is not a transient state on the potential energy surface. This may indicate that the D-pathway proposed by us is not available for the second water molecule, consistent with characteristics in the B family. It is probable that the reduction mechanisms of the A1 and B families are different. Thus, it can be thought that the A1 family should be at least distinguished from the B family.

## Supplementary Material

Table S1: Mulliken Charge (*ρ*) and Spin (*σ*) Populations (e) of Atoms and Groups in the Optimized Intermediates, 1–4.
Table S2: Mulliken Charge (*ρ*) and Spin (*σ*) Populations (e) of Atoms and Groups in the Optimized Intermediates, 4–7.
Table S3: Mulliken Charge (*ρ*) and Spin (*σ*) Populations (e) of Atoms and Groups in the Optimized Intermediates, 2 and 8–10.
Table S4: Mulliken Charge (*ρ*) and Spin (*σ*) Populations (e) of Atoms and Groups in the Optimized Intermediates, 10–13.
Table S5: Mulliken Charge (*ρ*) and Spin (*σ*) Populations (e) of Atoms and Groups in the Optimized Intermediates, 13–17.
Table S6: Mulliken Charge (*ρ*) and Spin (*σ*) Populations (e) of Atoms and Groups in the Optimized Intermediates, 17–19 and 1.
Figure S1: Optimized geometries of the Cu_B_ site for Cu(II) and Cu(I) without coordination of a H_2_O molecule.
Figure S2: Variations of properties with move of the H-atom from –CH_2_OH to the O_c_-atom of Tyr244. (A) Relative energy, (B) charge populations, (C) spin populations, and (D) atomic distances.
Figure S3: Geometries at *R* OH = 1.4 and 1.35 Å on the way that the H-atom moves from – CH_2_OH to the O_c_-atom of Tyr244.
Figure S4: Variations of relative energy with move of the H-atom from –CH_2_OH to the O_c_-2 atom of Tyr244.
Figure S5: Variations of (A) relative energy and (B) spin populations for formation of first H_2_O molecule in MV C*C*O.
Figure S6: Geometries at *R* OO = 1.6 and 1.7 Å on the way from 6 (*R* OO = 1.479 Å) to 7 (*R* OO = 2.631 Å).
Figure S7: Variations of (A) relative energy and (B) spin populations with move of the H-atom from the O-atom of Tyr244 to W_1_.
Figure S8: Variations of relative energy from 11 to 12 with movement of a proton from –CH_2_OH to Tyr244.
Figure S9: Variations of (A) relative energy and (B) spin populations for formation of first H_2_O molecule in FR CcO.
Figure S10: Geometries at *R* OH = 1.9 and 2.0 Å on the way that the O-O bond cleaves to produce the first H_2_O molecule in FR C*C*O.

## Figures and Tables

**Table 1:** Oxidation States of Electron Sites and Number of Electrons (*N*_e_) Relevant to O_2_ Reduction in FO, FR, and MV CcO.

	Cu_A_	Heme *a *	Heme *a*_3_	Cu_B_	N_e_
FO	II	III	III	II	0
FR	I	II	II	I	4
MV	II	III	II	I	2

**Table 2:** Total energies (au), expectation values of square of spin angular momentums (au), and relative energies (kcal/mol) of optimized intermediates.

Intermediates	(C,2S+1)^a^	*E*_total_	〈*S* ^2^〉	Δ*E*_rel_	
**1**	(1, 3)	−5548.568706	2.1488		
**2**	(1, 1)	−5698.927805	0.9297	0.0	26.7^b^
	(1, 3)	−5698.922264	2.0241	3.5	
**3**	(2, 1)	−5699.226658	0.9388	0.0	
**4**	(2, 1)	−5699.280053	1.0223	−33.5	
**5**	(3, 1)	−5699.492987	1.0247	0.0	
**6**	(3, 1)	−5699.501523	1.0280	−5.4	
**7**	(3, 1)	−5699.526079	2.0580	−20.8	
**8**	(0, 2)	−5699.056867	0.7749	81.0^c^	60.5^d^
**9**	(1, 2)	−5699.450706	0.7843	0.0	
**10**	(1, 2)	−5699.538816	0.7663	−55.3	
**11**	(2, 2)	−5699.846714	0.7672	0.0	
**12**	(2, 2)	−5699.870050	0.7720	−14.6	
**12a**	(2, 2)	−5699.866225	0.7715	−12.2	
**13**	(2, 2)	−5699.906221	1.7773	−37.3	
**14**	(2, 2)	−5776.364920	1.7725		
**15**	(1, 3)	−5776.635143	2.0192		
**16**	(2, 3)	−5777.019546	2.0295		
**17**	(3, 3)	−5777.261791	2.0402		
**18**	(3, 3)	−5624.389728	2.2087		
**19**	(2, 4)	−5624.742138	3.8743		

^a^(*C*, 2*S*+1) means (total charge, spin multiplicity).

^b^O_2_-binding energy: Δ*E*_rel_ = *E*(2) −* E*(1) − *E*(O_2_).

^c^Electron affinity of **2:**Δ*E*_rel_ = *E*(2) −* E*(8).

^d^O_2_^−^-binding energy: Δ*E*_rel_ = *E*(8) −* E*(1) − *E*(O_2_^−^).

**Table 3:** Interatomic distances (Å) of key atoms in the optimized intermediates^*a*^.

Intermediates	Fe-O_a_	O_a_-O_b_	O_b_-O (W_1_)	O_c_-O(W_1_)	O_d_-O (W_2_)	Cu-O (W_3_)	O_a_-O (W_3_)
**1 **				2.850	2.746		
**2 **	1.879	1.301	3.652	2.871	2.744		
**3 **	1.891	1.307	2.801	2.561	2.528		
**4 **	1.856	1.449	2.953	2.743	2.716		
**5 **	1.866	1.465	2.791	2.581	2.503		
**6 **	1.887	1.479	2.528	2.558	2.677		
**7 **	1.658	2.631	2.758	2.807	2.690		
**8 **	1.941	1.328	3.073	2.891	2.745		
**9 **	1.918	1.336	2.593	2.481	2.567		
**10 **	1.827	1.443	3.090	2.745	2.744		
**11 **	1.844	1.453	2.726	2.532	2.532		
**12 **	1.908	1.484	2.506	2.650	2.712		
**12a **	1.875	1.489	2.497	2.668	2.708		
**13 **	1.660	2.650	2.865	2.954	2.722		
**14 **	1.676	2.646	2.862	2.954	2.723	2.037	2.601
**15 **	1.841	2.514	2.713	2.967	2.750	1.904	2.804
**16 **	1.928	2.710	2.713	2.738	2.722	1.926	2.510
**17 **	2.057	2.582	2.670	2.701	2.697	2.068	2.742
**18 **				2.758	2.692	2.042	
**19 **				2.815	2.717	2.227	

^a^Oxygen symbols, O_a_, O_b_, Oc, and O_d_, are shown in Figure 2. O_a_ and O_b_ are oxygen atoms to be reduced to 2H_2_O, O_c_, and O_d_ are oxygen atoms of phenol (Tyr244) and −CH_2_OH, respectively.
