# Supplementary material for: B3LYP Study on Reduction Mechanisms from O2 to H2O at the Catalytic Sites of Fully Reduced and Mixed-Valence Bovine Cytochrome c Oxidases
Source: Bioinorg Chem Appl. 2010 Apr 6;2010:182804. doi: 10.1155/2010/182804 (PMC2852611; doi:10.1155/2010/182804)
Supplement: Supplementary file 1 — Table S1: Mulliken Charge (ρ) and Spin (σ) Populations (e) of Atoms and Groups in the Optimized Intermediates, 1–4. Table S2: Mulliken Charge (ρ) and Spin (σ) Populations (e) of Atoms and Groups in the Optimized Intermediates, 4–7. Table S3: Mulliken Charge (ρ) and Spin (σ) Populations (e) of Atoms and Groups in the Optimized Intermediates, 2 and 8–10. Table S4: Mulliken Charge (ρ) and Spin (σ) Populations (e) of Atoms and Groups in the Optimized Intermediates, 10–13. Table S5: Mulliken Charge (ρ) and Spin (σ) Populations (e) of Atoms and Groups in the Optimized Intermediates, 13–17. Table S6: Mulliken Charge (ρ) and Spin (σ) Populations (e) of Atoms and Groups in the Optimized Intermediates, 17–19 and 1. Figure S1: Optimized geometries of the CuB site for Cu(II) and Cu(I) without coordination of a H2O molecule. Figure S2: Variations of properties with move of the H-atom from –CH2OH to the Oc-atom of Tyr244. (A) Relative energy, (B) charge populations, (C) spin populations, and (D) atomic distances. Figure S3: Geometries at R OH = 1.4 and 1.35 Å on the way that the H-atom moves from – CH2OH to the Oc-atom of Tyr244. Figure S4: Variations of relative energy with move of the H-atom from –CH2OH to the Oc-2 atom of Tyr244. Figure S5: Variations of (A) relative energy and (B) spin populations for formation of first H2O molecule in MV CCO. Figure S6: Geometries at R OO = 1.6 and 1.7 Å on the way from 6 (R OO = 1.479 Å) to 7 (R OO = 2.631 Å). Figure S7: Variations of (A) relative energy and (B) spin populations with move of the H-atom from the O-atom of Tyr244 to W1. Figure S8: Variations of relative energy from 11 to 12 with movement of a proton from –CH2OH to Tyr244. Figure S9: Variations of (A) relative energy and (B) spin populations for formation of first H2O molecule in FR CcO. Figure S10: Geometries at R OH = 1.9 and 2.0 Å on the way that the O-O bond cleaves to produce the first H2O molecule in FR CCO. [file 182804.f1.pdf]

Supporting Information

for

B3LYP Study on Reduction Mechanisms from O_2 to H_2O at the Catalytic Sites of Fully Reduced and Mixed-Valence Bovine Cytochrome *c* Oxidases

Yasunori Yoshioka* and Masaki Mitani

Chemistry Department for Materials, Graduate School of Engineering, Mie University,

Kurima-machiya 1577, Tsu, Mie 514-8507, Japan

TABLE S1: Mulliken Charge (ρ) and Spin (σ) Populations (e) of Atoms and Groups in the Optimized Intermediates, 1- 4

Intermediates	**1**		**2**		**3**		**4**	
(C, 2S+1)[a]	(1, 3)		(1, 1)		(2, 1)		(2, 1)	
	ρ	σ	ρ	σ	ρ	σ	ρ	σ
Fe	1.504	2.151	1.664	1.062	1.672	1.055	1.674	0.964
Cu	0.694	0.001	0.738	-0.014	0.759	-0.039	1.062	-0.507
Por	-1.482	-0.259	-1.146	-0.056	-0.371	-0.055	-0.894	-0.332
H376	0.037	0.107	0.110	0.008	0.143	0.008	0.128	-0.005
H-T	0.092	0.000	0.062	-0.002	0.105	-0.001	0.148	-0.035
H290	0.026	-0.000	0.015	-0.001	0.030	-0.000	0.163	-0.108
H291	0.080	0.000	0.066	0.000	0.090	-0.001	0.180	-0.048
OO			-0.553	-0.996	-0.599	-0.959		
OOH							-0.561	0.071
W_1	-0.017	0.000	-0.010	-0.001	0.044	-0.007	0.028	0.000
W_2	0.066	0.000	0.066	0.000	0.128	0.000	0.073	0.000
Cu_B[b]	0.892	0.001	0.881	-0.017	0.984	-0.041	1.553	-0.698

[a] (C, 2S+1) means (total charge, spin multiplicity).

[b] $\rho(Cu_B) = \rho(Cu) + \rho(H290) + \rho(H291) + \rho(H\text{-}T)$ and $\sigma(Cu_B) = \sigma(Cu) + \sigma(H290) + \sigma(H291) + \sigma(H\text{-}T)$.

TABLE S2: Mulliken Charge (ρ) and Spin (σ) Populations (e) of Atoms and Groups in the Optimized Intermediates, 4 - 7

Intermediates	**4**		**5**		**6**		**7**	
(C, 2S+1)[a]	(2, 1)		(3, 1)		(3, 1)		(3, 1)	
	ρ	σ	ρ	σ	ρ	σ	ρ	σ
Fe	1.674	0.964	1.682	0.984	1.686	1.011	1.708	1.293
Cu	1.062	-0.507	1.062	-0.517	1.046	-0.495	1.100	-0.590
Por	-0.894	-0.332	-0.120	-0.314	-0.729	-0.350	-0.173	-1.079
H376	0.128	-0.005	0.168	-0.002	0.186	-0.002	0.173	-0.002
H-T	0.148	-0.035	0.210	-0.031	0.214	-0.030	0.249	-0.125
H290	0.163	-0.108	0.190	-0.128	0.189	-0.115	0.267	-0.211
H291	0.180	-0.048	0.206	-0.056	0.206	-0.054	0.226	-0.075
OOH	-0.561	0.071	-0.564	0.064	-0.561	0.035		
O							-0.644	0.785
H_2O							-0.004	0.005
W_1	0.028	0.000	0.026	0.000			0.015	-0.000
W_1H^+					0.677	0.000		
W_2	0.073	0.000	0.140	0.000	0.086	0.000	0.082	0.000
Cu_B[b]	1.553	-0.698	1.668	-0.732	1.655	-0.694	1.842	-1.001

[a] (C, 2S+1) means (total charge, spin multiplicity).

[b] $\rho(Cu_B) = \rho(Cu) + \rho(H290) + \rho(H291) + \rho(H\text{-}T)$ and $\sigma(Cu_B) = \sigma(Cu) + \sigma(H290) + \sigma(H291) + \sigma(H\text{-}T)$.

TABLE S3: Mulliken Charge (ρ) and Spin (σ) Populations (e) of Atoms and Groups in the Optimized Intermediates, 2 and 8 - 10

Intermediates	**2**		**8**		**9**		**10**	
(C, 2S+1)[a]	(1, 1)		(0, 2)		(1, 2)		(1, 2)	
	ρ	σ	ρ	σ	ρ	σ	ρ	σ
Fe	1.664	1.062	1.561	0.157	1.575	0.233	1.667	0.934
Cu	0.738	-0.014	0.740	0.002	0.748	0.005	0.750	-0.000
Por	-1.146	-0.056	-1.701	-0.108	-0.894	-0.114	-1.208	-0.055
H376	0.110	0.008	0.005	-0.007	0.042	-0.005	0.092	-0.002
H-T	0.062	-0.002	0.047	0.002	0.037	-0.001	0.057	-0.000
H290	0.015	-0.001	0.002	0.001	0.018	0.001	0.018	-0.000
H291	0.066	0.000	0.048	-0.000	0.066	-0.000	0.062	0.000
OO	-0.553	-0.996	-0.724	0.953	-0.743	0.875		
OOH							-0.523	0.124
W_1	-0.010	-0.001	-0.011	0.000	0.034	0.006	0.017	-0.000
W_2	0.066	0.000	0.033	-0.000	0.116	-0.000	0.067	0.000
Cu_B[b]	0.881	-0.017	0.838	0.005	0.869	0.005	0.887	-0.000

[a] (C, 2S+1) means (total charge, spin multiplicity).

[b] $\rho(Cu_B) = \rho(Cu) + \rho(H290) + \rho(H291) + \rho(H\text{-}T)$ and $\sigma(Cu_B) = \sigma(Cu) + \sigma(H290) + \sigma(H291) + \sigma(H\text{-}T)$.

TABLE S4: Mulliken Charge (ρ) and Spin (σ) Populations (e) of Atoms and Groups in the Optimized Intermediates, 10 - 13

Intermediates	**10**		**11**		**12**		**12a**		**13**	
$(C, 2S+1)^a$	(1, 2)		(2, 2)		(2, 2)		(2, 2)		(2, 2)	
	ρ	σ	ρ	σ	ρ	σ	ρ	σ	ρ	σ
Fe	1.667	0.934	1.683	0.970	1.691	1.026	1.684	1.015	1.710	1.304
Cu	0.750	-0.000	0.729	-0.000	0.749	-0.000	0.730	0.001	1.022	-0.486
Por	-1.208	-0.055	-0.413	-0.059	-1.032	-0.045	-1.013	-0.044	-0.813	-0.386
H376	0.092	-0.002	0.133	0.005	0.163	-0.002	0.164	-0.003	0.144	0.006
H-T	0.057	-0.000	0.123	-0.000	0.124	0.000	0.104	0.000	0.147	-0.040
H290	0.018	-0.000	0.058	-0.000	0.097	-0.000	0.036	0.000	0.186	-0.136
H291	0.062	0.000	0.106	-0.000	0.125	-0.000	0.093	-0.000	0.181	-0.049
OOH	-0.523	0.124	-0.583	0.084						
O					-0.457	0.024	-0.449	0.037	-0.650	0.774
H_2O^b					0.392	-0.003	0.494	-0.006	0.010	0.013
W_1	0.017	-0.000	0.038	0.000	0.072	-0.000	0.082	-0.000	-0.007	-0.000
W_2	0.067	0.000	0.126	0.000	0.075	0.000	0.074	0.000	0.071	0.000
$Cu_B{}^c$	0.887	-0.000	1.016	-0.000	1.095	-0.000	0.964	0.001	1.536	-0.711

[a] $(C, 2S+1)$ means (total charge, spin multiplicity).

[b] First H_2O produced.

[c] $\rho(Cu_B) = \rho(Cu) + \rho(H290) + \rho(H291) + \rho(H\text{-}T)$ and $\sigma(Cu_B) = \sigma(Cu) + \sigma(H290) + \sigma(H291) + \sigma(H\text{-}T)$.

TABLE S5: Mulliken Charge (ρ) and Spin (σ) Populations (e) of Atoms and Groups in the Optimized Intermediates, 13 - 17

Intermediates	**13**		**14**		**15**		**16**		**17**	
(C, 2S+1)[a]	(2, 2)		(2, 2)		(1, 3)		(2, 3)		(3, 3)	
	ρ	σ	ρ	σ	ρ	σ	ρ	σ	ρ	σ
Fe	1.710	1.304	1.751	1.459	1.693	0.971	1.709	1.027	1.685	1.024
Cu	1.022	-0.486	1.153	-0.637	1.097	0.559	1.127	0.599	1.132	0.622
Por	-0.813	-0.386	-0.971	-0.106	-1.198	-0.066	-1.050	-0.049	-0.923	-0.023
H376	0.144	0.006	0.166	0.013	0.119	0.015	0.181	0.011	0.238	0.003
H-T	0.147	-0.040	0.143	-0.082	0.093	0.063	0.143	0.067	0.191	0.087
H290	0.186	-0.136	0.194	-0.112	0.100	0.035	0.137	0.050	0.218	0.123
H291	0.181	-0.049	0.194	-0.101	0.141	0.084	0.175	0.098	0.221	0.113
O	-0.650	0.774	-0.740	0.619						
OH(Fe)					-0.538	0.076				
H_2O[b]	0.010	0.013	0.009	0.013	-0.009	0.003	0.012	-0.000	0.019	0.000
H_2O[c]							-0.019	0.013	0.015	-0.004
W_1	-0.007	-0.000	-0.006	-0.000	-0.044	0.000	0.029	0.000	0.046	0.000
W_2	0.071	0.000	0.072	0.000	0.065	0.000	0.072	0.000	0.078	-0.000
W_3			0.035	-0.065					0.079	0.056
OH(W_3)					-0.518	0.260	-0.517	0.185		
Cu_B[d]	1.536	-0.711	1.684	-0.932	1.431	0.741	1.582	0.814	1.761	0.945

[a] (C, 2S+1) means (total charge, spin multiplicity).

[b] First H_2O produced.

[c] Second H_2O produced.

[d] $\rho(Cu_B) = \rho(Cu) + \rho(H290) + \rho(H291) + \rho(H\text{-}T)$ and $\sigma(Cu_B) = \sigma(Cu) + \sigma(H290) + \sigma(H291) + \sigma(H\text{-}T)$.

TABLE S6: Mulliken Charge (ρ) and Spin (σ) Populations (e) of Atoms and Groups in the Optimized Intermediates, 17 – 19 and 1

Intermediates	**17**		**18**		**19**		**1**	
$(C, 2S+1)^a$	(3, 3)		(3, 3)		(2, 4)		(1, 3)	
	ρ	σ	ρ	σ	ρ	σ	ρ	σ
Fe	1.685	1.024	1.585	1.093	1.596	2.538	1.504	2.151
Cu	1.132	0.622	1.134	0.614	0.946	0.340	0.694	0.001
Por	-0.923	-0.023	-0.808	-0.072	-1.107	-0.141	-1.482	-0.259
H376	0.238	0.003	0.270	-0.021	0.107	0.148	0.037	0.107
H-T	0.190	0.087	0.200	0.092	0.116	0.021	0.092	0.000
H290	0.218	0.123	0.240	0.130	0.123	0.047	0.026	-0.000
H291	0.221	0.113	0.213	0.108	0.122	0.034	0.080	0.000
H_2O^b	0.019	0.000						
H_2O^c	0.015	-0.004						
W_1	0.046	0.000	-0.004	0.001	-0.011	0.000	-0.017	0.000
W_2	0.078	-0.000	0.082	0.000	0.073	0.000	0.066	0.000
W_3	0.079	0.056	0.086	0.055	0.036	0.013		
Cu_B^d	1.761	0.945	1.787	0.944	1.306	0.441	0.892	0.001

[a] $(C, 2S+1)$ means (total charge, spin multiplicity).

[b] First H_2O produced.

[c] Second H_2O produced.

[d] $\rho(Cu_B) = \rho(Cu) + \rho(H290) + \rho(H291) + \rho(H\text{-}T)$ and $\sigma(Cu_B) = \sigma(Cu) + \sigma(H290) + \sigma(H291) + \sigma(H\text{-}T)$.

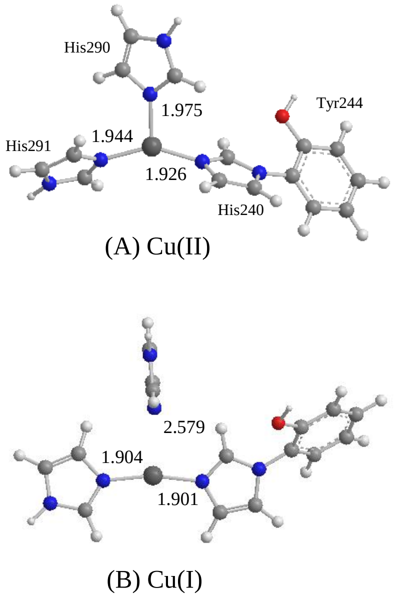

FIGURE S1: Y. Yoshioka et al.

FIGURE S1: Optimized geometries of the Cu_B site for Cu(II) and Cu(I) without coordination of a H_2O molecule.

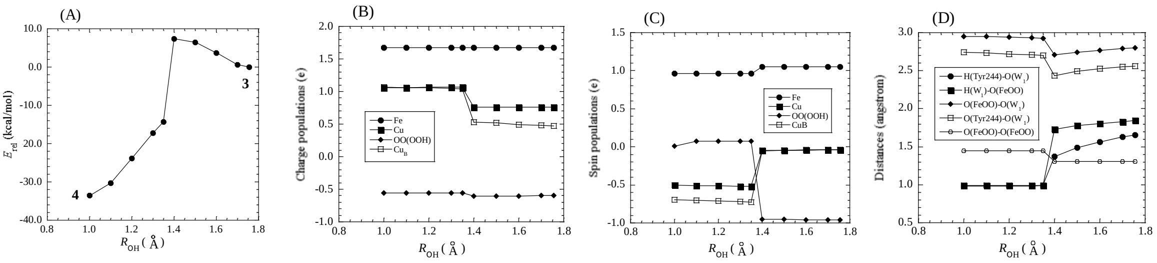

FIGURE S2: Y. Yoshioka et al.

FIGURE S2: Variations of properties with move of the H-atom from –CH_2OH to the O_c-atom of Tyr244. (A) Relative energy, (B) charge populations, (C) spin populations, and (D) atomic distances.

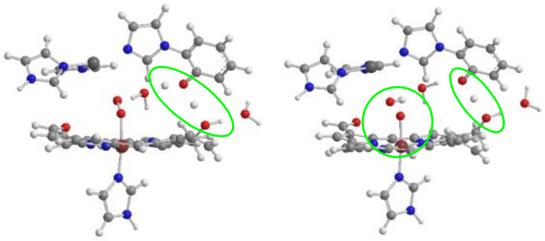

3a (R_{OH} = 1.4) **4a** (R_{OH} = 1.35)

FIGURE S3: Y. Yoshioka et al.

FIGURE S3: Geometries at R_{OH} = 1.4 and 1.35 Å on the way that the H-atom moves from –CH_2OH to the O_c-atom of Tyr244.

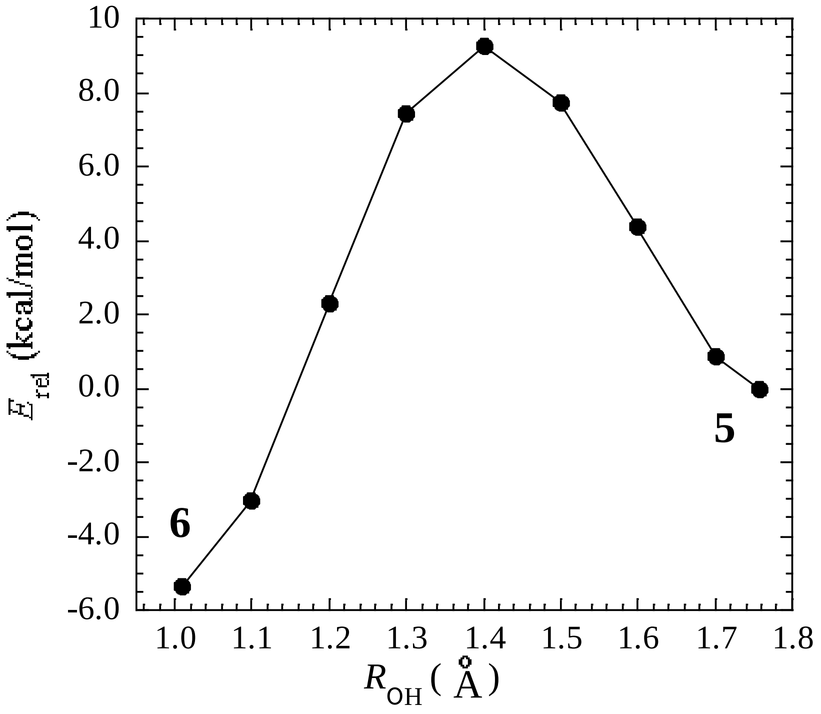

FIGURE S4: Y. Yoshioka et al.

FIGURE S4: Variations of relative energy with move of the H-atom from –CH_2OH to the O_c-atom of Tyr244.

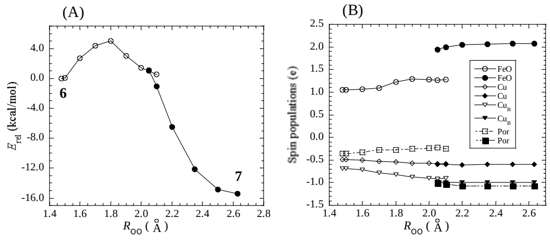

FIGURE S5: Y. Yoshioka et al.

FIGURE S5: Variations of (A) relative energy and (B) spin populations for formation of first H_2O molecule in MV C*c*O.

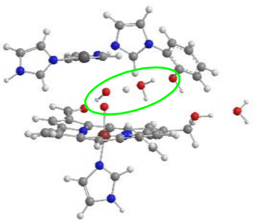

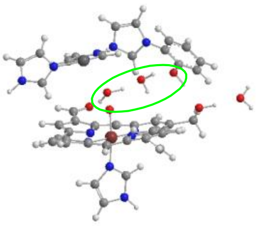

R_{OO} = 1.6 (3, 1) R_{OO} = 1.7 (3, 1)

FIGURE S6: Y. Yoshioka et al.

FIGURE S6: Geometries at R_{OO} = 1.6 and 1.7 Å on the way from **6** (R_{OO} = 1.479 Å) to **7** (R_{OO} = 2.631 Å).

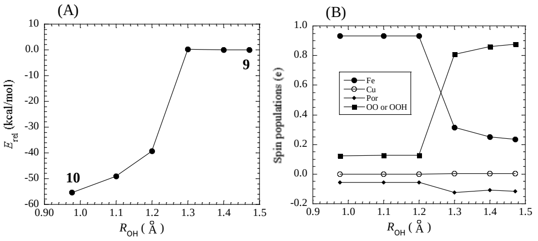

FIGURE S7: Y. Yoshioka et al.

FIGURE S7: Variations of (A) relative energy and (B) spin populations with move of the H-atom from the O-atom of Tyr244 to W_1.

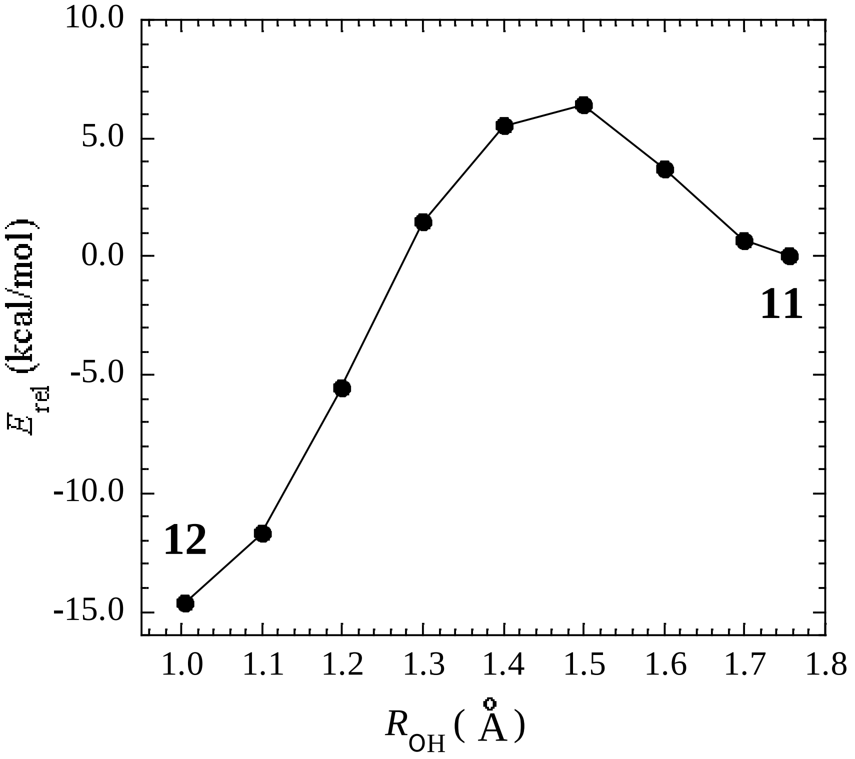

FIGURE S8: Y. Yoshioka et al.

FIGURE S8: Variations of relative energy from **11** to **12** with movement of a proton from –CH_2OH to Tyr244.

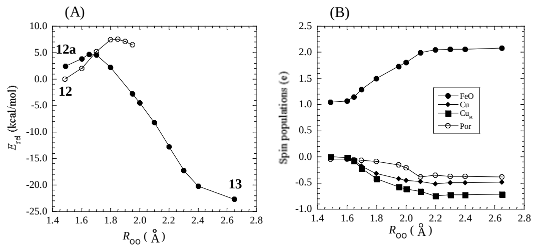

FIGURE S9: Y. Yoshioka et al.

FIGURE S9: Variations of (A) relative energy and (B) spin populations for formation of first H_2O molecule in FR C*c*O.

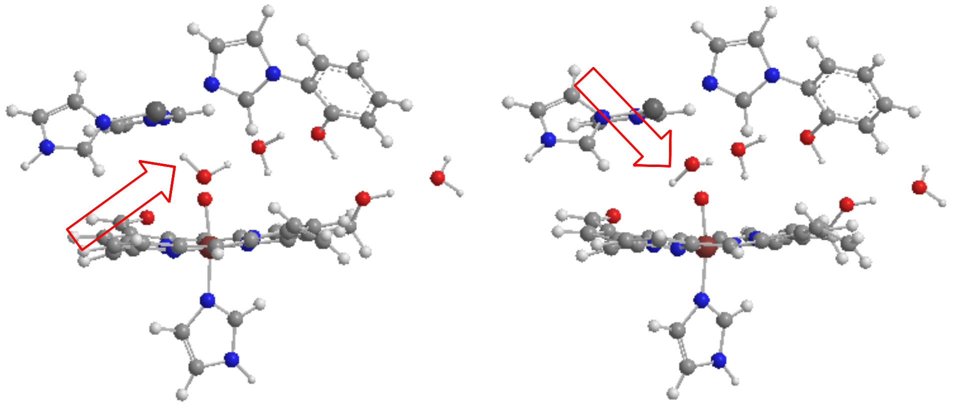

R_{OO} = 1.9 R_{OO} = 2.0

FIGURE S10: Y. Yoshioka et al.

FIGURE S10: Geometries at R_{OH} = 1.9 and 2.0 Å on the way that the O-O bond cleaves to produce the first H_2O molecule in FR C*c*O.
